# Bone Fragility in High Fat Diet-induced Obesity is Partially Independent of Type 2 Diabetes in Mice

**DOI:** 10.1007/s00223-024-01252-x

**Published:** 2024-07-16

**Authors:** Sasidhar Uppuganti, Amy Creecy, Daniel Fernandes, Kate Garrett, Kara Donovan, Rafay Ahmed, Paul Voziyan, Elizabeth Rendina-Ruedy, Jeffry S. Nyman

**Affiliations:** 1 Department of Orthopaedic Surgery, Vanderbilt University Medical Center, Medical Center East, South Tower, 1215 21st Ave. S., Suite 4200, Nashville, TN 37232 USA; 2 Vanderbilt Center for Bone Biology, Vanderbilt University Medical Center, 2215B Garland Ave., Nashville, TN 37212 USA; 3 Department of Orthopaedic Surgery, Indiana University School of Medicine, 550 N. University Blvd, Indianapolis, IN 46202 USA; 4 Department of Biomedical Engineering, Vanderbilt University, 5824 Stevenson Center, Nashville, TN 37232 USA; 5 Department of Medicine, Division of Clinical Pharmacology, Vanderbilt University Medical Center, Nashville, TN 37232 USA; 6 United States Department of Veterans Affairs, Tennessee Valley Healthcare System, 1310 24th Ave. S., Nashville, TN 37212 USA; 7 Department of Molecular Physiology and Biophysics, Vanderbilt University Medical Center, 2215 Garland Ave., Nashville, TN 37232 USA

**Keywords:** Type 2 Diabetes, Bone mass, Bone quality, Diet-induced obesity, Mechanical properties, Advanced glycation end-products

## Abstract

Obesity and type 2 diabetes (T2D) are risk factors for fragility fractures. It is unknown whether this elevated risk is due to a diet favoring obesity or the diabetes that often occurs with obesity. Therefore, we hypothesized that the fracture resistance of bone is lower in mice fed with a high fat diet (45% kcal; HFD) than in mice that fed on a similar, control diet (10% kcal; LFD), regardless of whether the mice developed overt T2D. Sixteen-week-old, male NON/ShiLtJ mice (resistant to T2D) and age-matched, male NONcNZO10/LtJ (prone to T2D) received a control LFD or HFD for 21 weeks. HFD increased the bodyweight to a greater extent in the ShiLtJ mice compared to the NZO10 mice, while blood glucose levels were significantly higher in NZO10 than in ShiLtJ mice. As such, the glycated hemoglobin A1c (HbA1c) levels exceeded 10% in NZO10 mice, but it remained below 6% in ShiLtJ mice. Diet did not affect HbA1c. HFD lowered trabecular number and bone volume fraction of the distal femur metaphysis (micro-computed tomography or μCT) in both strains. For the femur mid-diaphysis, HFD significantly reduced the yield moment (mechanical testing by three-point bending) in both strains but did not affect cross-sectional bone area, cortical thickness, nor cortical tissue mineral density (μCT). Furthermore, the effect of diet on yield moment was independent of the structural resistance of the femur mid-diaphysis suggesting a negative effect of HFD on characteristics of the bone matrix. However, neither Raman spectroscopy nor assays of advanced glycation end-products identified how HFD affected the matrix. HFD also lowered the resistance of cortical bone to crack growth in only the diabetic NZO10 mice (fracture toughness testing of other femur), while HFD reduced the ultimate force of the L6 vertebra in both strains (compression testing). In conclusion, the HFD-related decrease in bone strength can occur in mice resistant and prone to diabetes indicating that a diet high in fat deleteriously affects bone without necessarily causing hyperglycemia.

**Supplementary Information:**

The online version contains supplementary material available at 10.1007/s00223-024-01252-x.

## Introduction

Based on the US National Health and Nutrition Examination Survey (NHANES), the prevalence of obesity—as defined by either body mass index (BMI) or relative fat mass—has steadily increased between 2000 and 2020 in both men and women [1]. Analysis of trends in the NHANES data also revealed that the prevalence of diabetes has also increased over the past several decades [2]. Along with obesity being a major risk factor for developing type 2 diabetes (T2D), it portends complications arising from this disease, thereby increasing morbidity and mortality [3]. Moreover, as the duration of T2D increases, patients develop many different complications such as cardiovascular disease, kidney disease, and neuropathy. Another comorbidity of T2D is an elevated fracture risk that is disproportionate to the patient’s areal bone mineral density (aBMD), the clinical standard for the assessment of osteoporosis [4]. In effect, an adult with T2D has a higher risk of suffering a fragility fracture of the hip than an adult without diabetes with the same measurement of hip aBMD [5]. While review articles have postulated different mechanisms to explain bone fragility in T2D [6], there is no clear therapeutic option for preventing fractures in T2D [7]. This is problematic because the number of fragility fractures is likely to increase in the coming years along with the increasing prevalence of obesity worldwide [8].

One barrier to the development and assessment of therapies to treat diabetic bone disease is a rodent model of T2D that mimics both hyperglycemia, stable weight (i.e., no loss in body mass due to uncontrolled diabetes), and a decrease in the fracture resistance of bone that cannot be solely explained by a reduction in aBMD or volumetric BMD. There are multiple rodent models of T2D for which the bone phenotype has been reported, though measurements of bone strength and toughness in these models are less documented than measurements of bone morphology and density [9]. Perhaps one attractive model to investigate the effect of diabetes on bone is feeding mice a high fat diet (HFD) to induce obesity and impair glucose tolerance of the animal since obesity and a diet high in fat are common features of T2D [10, 11]. Of pre-clinical studies published before 2024 that report differences in various bone properties between mice fed a HFD and mice fed a control diet (Table S1), approximately half included mechanical testing of bone to determine differences in bone strength, namely *quasi*-static, load-to-failure, three-point or four-point bending tests of the tibia or femur [12–29], but even fewer studies attempted to infer an effect of diet-induced obesity (DIO) on material properties (i.e., independent of structure and bone mass) of cortical bone by mechanical tests [12, 13, 22, 24, 27, 28]. To the best of knowledge, these studies primarily fed growing (≤15-weeks.), male, C57BL/6 mice a HFD or LFD with variable duration (Table S1). Only 1 study reported differences in compressive strength of lumbar vertebral body (VB), a trabecular rich site [14].

In addition to the paucity of information about the effect of DIO on the fracture resistance of bone, we do not know if the HFD alone or the resulting glucose intolerance is responsible for bone fragility. Most published mouse studies using DIO to investigate diabetic effects on bone involved C57BL/6 mice (sub-strain, J or N, not always being clear) since this widely available inbred strain becomes glucose intolerant, especially when fed a HFD starting at a young age (3–6 weeks-old) [30]. Since most DIO studies started HFD in growing mice, the effect of T2D on bone in adult mice is less clear. In the few published studies that switched from standard rodent chow to a purified diet with different fat content at or near skeletal maturity (i.e., 15-weeks, 20-weeks, or 42-weeks of age), circulating glucose levels were significantly higher in the C57BL/6 mice fed HFD compared to C57BL/6 mice fed LFD [13, 14, 31]. Upon consuming either diet for 12 weeks and 16 weeks prior to euthanasia, the ultimate compressive force of the L3 VB [14] and the ultimate stress of the femur mid-diaphysis in bending [13] was lower in the HFD than in the LFD group, respectively. Like most DIO studies (Table S1) though, it is unclear if this loss in fracture resistance of bone was due to the high fat content, typically lard, or disturbance in glucose metabolism that the HFD caused. Except for 2 DIO mouse studies [24, 27], biochemical assessment of advanced glycation end-products (AGEs) in the bone tissue was not reported. The reporting of elevated blood glucose is variable among DIO studies (Table S1); and moreover, these studies did not assess the severity of T2D using glycated hemoglobin A1c (HbA1c). Therefore, it is an open question as to whether glucose-mediated changes to the matrix and/or bone metabolism are responsible for the bone fragility in the DIO model of T2D.

As discussed in the review of pre-clinical models of T2D-related bone fragility by Fajardo et al. [9], there is a need to characterize the bone phenotype of prevalent rodent models of T2D. The NONcNZO10/LtJ mouse from The Jackson Laboratory (Bar Harbor, ME, USA) is one such model of T2D that has not been characterized with respect to how hyperglycemia affects the fracture resistance of bone. This recombinant congenic strain is the result of breeding New Zealand Obese (NZO/HiLtJ) mice with non-obese, non-diabetic (NON/LtJ) mice and then backcrossing the F1 offspring onto the NON/LtJ background such that mice with known chromosomal locations of quantitative trait loci for diabetes from both strains were selected [32]. The resulting NONcNZO10/LtJ (NZO10) mouse develops obesity, insulin resistance, and hyperglycemia compared to NON/LtJ mice, especially if the mice are male [33]. In one of the first published reports using the NZO10 mouse model of spontaneous T2D [34], cutaneous wound healing of a full thickness incision on the dorsum was impaired relative to the recommended male control mice, presently known as NON/ShiLtJ (ShiLtJ) strain [34, 35]. That is, after 14 days of healing, the tensile strength of the wound was significantly lower in the NZO10 than in the ShiLtJ mice, though the difference was not as great as observed in the streptozotocin and db/db models of type 1 diabetes and T2D, respectively. Desirable features of this polygenic model of T2D include: transition to a diabetic state occurs close to skeletal maturity (~ 13-weeks of age), hyperphagia is not an issue, and NZO10 mice do not exhibit hypercorticism [35].

To investigate whether high fat diet alone or elevated glucose levels (> 250 mg/dl) affect the fracture resistance of bone, we fed adult male mice resistant (ShiLtJ) and susceptible (NZO10) to T2D either a high fat diet (45% kcal from fat) or a similar diet with lower fat content (10% kcal from fat) for 21 weeks. We hypothesized that HFD lowers the bending strength of cortical bone, the compressive strength of trabecular bone, and the fracture toughness of cortical bone, irrespective of diabetes. Bones were also harvested from ShiLtJ and NZO10 mice at 15-weeks to determine if differences in the bone phenotype exist before diet manipulation. By providing evidence for the stated hypothesis, mechanisms by which HFD lowers the fracture resistance of bone in mice without T2D (e.g., diet-induced systemic inflammation favors bone loss) and in mice with T2D (e.g., diabetes-related AGE accumulation-induced vs. diet-induced systemic inflammation favors bone loss) can be tested.

## Materials and Methods

### Study Design

Forty-five male NONcNZO10/LtJ (NZO10) mice (Jax Strain 004456) and 36 male NON/ShiLtJ (ShiLtJ) mice (Jax Strain 002423), the recommended control strain, were obtained from The Jackson Laboratory (Bar Harbor, ME, USA). We ordered a higher number of NZO10 mice because we anticipated that some of the NZO10 mice would not develop hyperglycemia. Arriving at 6–8 weeks of age after weaning on LabDiet® 5K20, the mice were fed a standard in-house rodent chow (LabDiet® 5L0D) until 16 weeks of age. Six ShiLtJ and eight NZO10 mice were euthanized at 15 weeks of age. The remaining mice were switched to Research Diets, Inc. (New Brunswick, NJ) D12450H (LFD: 10% kcal from fat) or Research Diets, Inc. D12451 (45% kcal from fat) at 16 weeks of age. Food and water were provided ad libitum throughout the study, and the mice experienced a standard 12-h light–dark cycle. Each diet was changed out once a week per the manufacturer’s recommendations. Body mass and non-fasting glucose measurements were measured weekly until 20 weeks when these measurements were switched to biweekly. All procedures followed a protocol approved by the IACUC at Vanderbilt University Medical Center.

Five ShiLtJ mice (3 on HFD) and two NZO10 (1 on HFD) had to be euthanized early because they developed an infection, at which point, we started housing the mice in sterilized cages. Another 3 NZO10 mice (2 on HFD) had to be euthanized before reaching 37 weeks of age because the mice were not thriving likely due to uncontrolled diabetes. Six ShiLtJ and 8 NZO10 mice were euthanized by cardiac exsanguination and cervical dislocation at 15 weeks of age to determine if differences in bone existed before diet manipulation. All other mice were euthanized by cardiac exsanguination and cervical dislocation at 37 weeks of age giving 4 groups: ShiLtJ-LFD (*n* = 13), ShiLtJ-HFD (*n* = 12), NZO10-LFD (*n* = 15), and NZO10-HFD (*n* = 17). Blood was then collected for measurements of HbA1c by Bayer DCA 2000 system at the Vanderbilt Mouse Metabolic Phenotyping Center. Upon harvesting both femurs and the L5-L6 VBs, they were immersed in phosphate buffered saline (PBS) and stored at − 20 °C until further testing.

### Micro-Computed Tomography (μCT) Evaluations of Femur and 6th Lumbar Vertebra

After thawing each femur and VB to room temperature, the samples were placed in a foam mold for stability inside a μCT tube holder (left intact femur: p/n U50821 Ø 6.0 mm; right notched femur, and L6 VB: p/n U50822 Ø 9.0 mm; Scanco Medical AG, Brüttisellen, Switzerland) and then immersed in PBS. Using a μCT50 scanner (Scanco Medical AG, Brüttisellen, Switzerland), the distal femur metaphysis (DFM) and mid-diaphysis of the left, intact femur (FMD) as well as the mid-diaphysis of the right (notched) femur were scanned following our previously published settings [36–38] that provide images with an isotropic voxel size of 6 μm (Table S2). Following our previously published scan settings [38, 39], the entire L6 VB was imaged at an isotropic voxel size of 12 μm. Following image reconstruction, we evaluated each region of interest (ROI)—DFM (2.7 mm in axial length terminating at ~ 0.3 mm above the growth plate), FMD (1.86 mm in length centered between the condylar fossa and the femoral neck), and VB (trabecular bone and cortical shell between the cranial and caudal end-plates)—as described previously by us for trabecular micro-architecture, cortical micro-structure, and tissue mineral density. The images of the micro-notch in the right femurs were analyzed to determine the angle (Fig. S1).

### Raman Spectroscopy Analysis of Notched Femur

The notched right femurs (see [36, 37] for details on the micro-notching procedure) were thawed to room temperature prior to their analysis using Raman micro-spectroscopy (InVia™ Raman microscope, Renishaw inc., Hoffman Estates, IL) to assess the bone composition. Each sample was carefully cleaned using a KimWipe™ and secured onto a microscope slide using sculpting putty (Premo! Sculpey, Polyform Products Co., Elk Grove Village, IL) such that the anterior surface (away from the notch) faced the microscope objective. After hydrating the sample, at the proximal and distal ends, the slide was secured inside the Raman microscope under a 20X objective (*NA* = 0.40). Using the Renishaw software (WiRE 4.2), a region on the anterior surface was montaged and 8 individual data points along the midshaft of the femur (spaced ~ 250–300 µm apart) were added to the measurement queue after adjusting their x, y and z-coordinates. The spectral acquisition settings were as follows: 830 nm laser (line focus), power ~ 35 mW, exposure time of 5 s, and average of 20 accumulations. Spectra were processed as described previously [37]. Briefly, the 8 raw Raman spectra per bone specimen were averaged to maximize the signal-to-noise. Then, background fluorescence was removed from all averaged spectra by subtracting a 5th-order polynomial function from the base of the raw spectra (Fig. S2). Next, the averaged spectra were smoothed to minimize noise using a proprietary de-noising (D-n) algorithm provided by the LabSpec software (v5.78.24, Horiba Jobin Yvon, Edison, NJ). From the averaged and de-noised spectrum per bone sample, the different Raman peak ratios were calculated using a custom script in Matlab r2018 (Mathworks inc., Natick, MA). Peak intensity ratios were calculated for ʋ_1_PO_4_^3−^/Amide I, ʋ_1_PO_4_^3−^/Amide III, ʋ_1_PO_4_^3−^/CH_2_-wag, ʋ_1_PO_4_^3−^/Proline, Hydroxyproline/Proline, and CO_3_^2−^/ʋ_1_PO_4_^3−^ (Fig. S2). Band area ratios were calculated for GAGs/ʋ_1_PO_4_^3−^, where wavenumber ranges of integration were as follows: 1365 cm^−1^ to 1386 cm^−1^ (GAGs), 925 cm^−1^ to 988 cm^−1^ (ʋ_1_PO_4_^3−^), 1492 cm^−1^ to 1503 cm^−1^ (PEN), 1433 cm^−1^ to 1490 cm^−1^ (CH_2_-wag), 1147 cm^−1^ to 1153 cm^−1^ (CML). Crystallinity was calculated using inverse of full-width at half maximum of ʋ_1_PO_4_^3−^ peak.

### Mechanical Testing of Intact Femur, Micro-Notched Femur, and VBs

After thawing the femurs to room temperature, total length and anterior–posterior (a-p) width of each bone was measured using digital calipers. Next, the anterior side of the left, intact femur was positioned on the lower supports (span = 7.0 mm, 7.5 mm, 8.0 mm, 8.5 mm, or 9.0 mm based on a-p width) with the medial side facing forward. Upon applying a pre-load of 0.2 N to the hydrated femur, the mid-diaphysis (i.e., three-point bending) was loaded at 3 mm/min until failure (Instron Dynamight 8841, Instron corporation, Norwood, MA). Each right, notched femur was centered with the micro-notch on the posterior side facing down and the medial side facing forward, on the lower span supports. The span of the three-point bending fixture was ~ 4 times the a-p width at the mid-diaphysis. The anterior side of the mid-diaphysis was loaded at 0.5 mm/min such that a crack propagated from the micro-notch (Fig. S3).

The dissected L6 VB was trimmed at the end-plates, using a scalpel blade, to expose the bony surface. Also, the facets were snipped using surgical scissors before the compression tests. The VB was hydrated with PBS and placed in between rigid, stainless steel compression platens with a pre-load force of 2N. The bottom compression platen was custom designed to provide moment-relief and favor axial compression of the VB. The VBs were loaded to failure at 3 mm/min (Instron Dynamight 8841, Instron corporation, Norwood, MA). All force data from the 100 N load cell and displacement data from the LVDT during each mechanical test were acquired at a sampling rate of 50 Hz, and moment vs. span-adjust displacement was processed as previously described by us [36, 37].

### Analysis of Fluorescent Advanced Glycation End-Products (fAGEs) and Collagen Crosslinks

After fracture toughness testing, each right femur (fAGE) was demineralized in 20% EDTA at 4 °C for 5 weeks. A right tibia (pentosidine) from selected mice was similarly demineralized. The samples were then dehydrated for 24 h at room temperature under a vacuum and then hydrolyzed in 6 N HCl at 110 °C for 20 h. After hydrolysis, the hydrochloric acid was evaporated using a SpeedVac Concentrator System (ThermoFisher) with a cold trap. Next, each hydrolysate was resuspended in HPLC-grade water and centrifuged at 15,000 g and 4 °C for 20 min. Supernatant aliquots corresponding to ∼1 mg of dry bone were used for each assay.

The pentosidine crosslink (PEN) concentration was measured using a high-performance liquid chromatography (HPLC) system (Agilent 1260 Infinity, Agilent Technologies, Santa Clara, CA) equipped with a Spherisorb 4.6 × 150 mm column (Waters Co, Milford, MA). The HPLC conditions were as follows: buffer A was 0.22% HFBA, buffer B was 100% acetonitrile, and the gradient program was 0.00–4.50 min, 13% B; 4.75–10.00 min, 17% B; 10.25–16.00 min 25% B; 16.75–21.00 min, 100% B; and 21.25–26.00 min, 13% B. The PEN was quantified using fluorescent detector at *λ*_ex_/*λ*_em_ = 328 nm/378 nm, a PEN standard (Cayman Chemical, Ann Arbor, MI), and pyridoxine as an internal standard (Sigma, St. Louis, MO). The PEN values were calculated based on the standard curve and normalized to collagen levels measured in the same samples.

A known fraction of each hydrolysate and quinine sulphate standards were dissolved in 0.1 M H_2_SO_4_ and analyzed as previously described [40]. Briefly, 100 μL of standard and sample solutions was pipetted in duplicate into a 96-well plate. Fluorescence was measured at λ_ex_/λ_em_ = 370/440 nm using BioTek Synergy H1 microplate reader. The levels of fAGEs were calculated based on the standard curve and normalized to collagen levels measured in the same samples.

Hydroxyproline (Hyp) levels were measured by a colorimetric assay as described in Stoilov I et al. [41]. Briefly, the assay was performed in 96-well plates following the addition of the oxidizer and Ehrlich’s solution to Hyp standards and the samples. The color was developed upon the incubation of the plates at 65 °C for 20 min and consequent rapid cooling. The measurements were taken at 550 nm using Synergy H1 microplate reader (BioTek Instruments, Winooski, VT). Collagen levels were calculated based on 290 Hyp residues per triple helical collagen molecule [42]. This colorimetric assay has been previously validated using HPLC [43].

### Statistical Analysis

To determine if body mass (BM) and blood glucose (BG) varied over time before and after the change from standard chow to a purified diet with different fat content, we used a mixed effects model in which BM and BG were repeated measures, mouse was matched, and age, strain, and their interaction were fixed effects (before diet change) or age, strain, diet, and their interactions were fixed effects (after diet change). This allowed for missing measurements because the variance between mice is estimated (Prism 10, GraphPad Software, LLC, Boston, MA). For end-point measurements, we used two-way ANOVA to determine if strain, diet, or their interaction significantly affected properties. In the event that the residuals in the two-way ANOVA did not pass the Spearman’s test for heteroscedasticity and/or the Anderson–Darling test for normality, we used either an ordinary one-way ANOVA if parametric assumptions were valid or Welch’s ANOVA if equal variance among the groups could not be assumed or the Kruskal–Wallis test if the residuals didn’t pass the aforementioned normality test to determine whether there was a significant difference among the 4 groups (Prism 10, GraphPad Software, LLC, Boston, MA). When there was a significant difference or a significant main effect, pair-wise comparisons included t-tests, Welch’s t-test, or Mann–Whitney U test (as appropriate for each group) in which the p-values were adjusted by the Holm-Šídák technique within diet or within strain (adj-p = 1 − (1 − lowest *p*-value)^2^ and adj-p = 1 − (1 − highest *p*-value)^1^). We did not compare ShiLtJ-LFD vs. NZO10-HFD nor ShiLtJ-HFD vs. NZO10-LFD. For selected bone properties in which differences in body mass could explain the effect of diet or strain, we used a general linear regression model in which diet and strain were categorical variables and body mass was a continuous variable (Prism 10, GraphPad Software, LLC, Boston, MA). Lastly, the Mann–Whitney test was used to determine if differences in bone properties between strains were significant at 15-weeks of age (i.e., before diet manipulation).

## Results

### High Fat Diet Increased Body Mass in Both Strains of Mice but Caused Persistent Diabetes in Only NONcNZO10/LtJ Mice

**Fig. 1:**
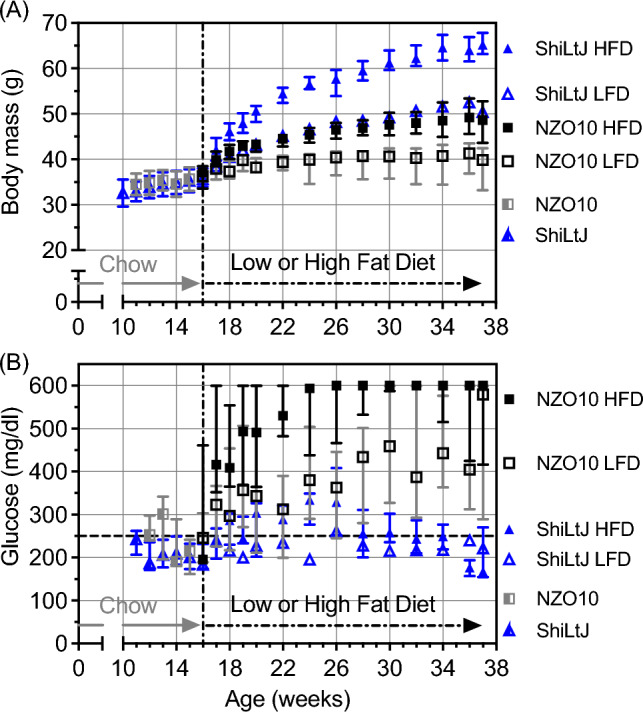
Age-related changes in body mass and non-fasting glucose before and after switching male mice from standard chow to 1 of 2 purified diets (medial ± IQR). **A** While the mice consumed a standard rodent chow, there were no pronounced differences in body mass between the 2 strains. When the mice started to consume a synthetic diet at 16 weeks of age, high fat increased body mass, but this depended on the strain. **B** From 11 to 16 weeks of age, a few mice had circulating levels above 250 mg/dl, but this was not consistent as they matured. Corresponding to the consumption of the high fat diet, all NONcNZ010/LtJ mice had glucose levels well above 250 mg/dl, while glucose levels increased for some NON/ShiLtJ mice

While consuming standard chow from 12 to 16-weeks, body mass (BM) did not depend on strain (Table 1), and so the initial BM was not significantly different between ShiLtJ and NZO10 mice (Table 2). Upon switching from standard rodent chow to a purified diet, mice on the HFD gained considerably more BM than mice on the LFD within each strain (Fig. 1A). This age-related weight gain significantly depended on strain and diet (Table 1) such that NON/ShiLtJ (ShiLtJ) mice gained the most BM by 37-weeks of age and that weight gain in NONcNZO10/LtJ (NZO10) mice on HFD was like the weight gain in ShiLtJ mice on LFD (Fig. 1A). As intended, initial BM did not vary between diet groups prior to switching the chow to LFD or HFD (Table 2). At the time of euthanasia, ShiLtJ mice weighed more than NZO10 mice within each diet group (Table 2).

**Table 1:** P-values from ANOVAs in which blood glucose (BG) and body mass were repeated measures before and after switching from standard chow to low or high fat diet. Terms were not included (NI) if not relevant (i.e., before switching to diet)

Fixed effects (mixed model^a^):	Age	Diet	Strain	Age × diet	Age × strain	Strain × diet	Age × Strain x diet
Body mass from 12-to-16-weeks	< 0.0001	NI	0.7643	NI	0.6674	NI	NI
BG from 12-to-16-weeks	0.2173	NI	0.0053	NI	0.0344	NI	NI
Body mass from 16-to-37-weeks	< 0.0001	< 0.0001	< 0.0001	< 0.0001	< 0.0001	0.5761	0.3302
BG from 16-to-37-weeks	< 0.0001	0.0002	< 0.0001	< 0.0001	< 0.0001	0.0544	0.1784

^a^Mouse modeled as a random effect

**Table 2:** Differences in body mass, glucose, and HbA1c between 4 groups of mice. Initial and final body mass (BM) and non-fasting blood glucose (BG) were the average of 15-to-16-weeks and 36-to-37-weeks of age, respectively (n = 10–17/group). HbA1c was determined at 37-weeks (n = 10/group)

		ANOVA^a^	ShiLtJ							Diet^b^	NZO10						Diet^b^	Strain	effect^b^
Property	Units	*p*-value	LFD		vs		HFD			effect	LFD		vs		HFD		effect	w/in LFD	w/in HFD
Initial BM	(g)	0.9720	35.6	±	2.2	35.5		±	3.1	N/A	35.8	±	2.4	35.9	±	2.8	N/A	N/A	N/A
Final BM	(g)	< 0.0001^†^	51.3	±	2.8	65.7		±	3.0^c^	< 0.0001	39.0	±	4.9	47.9	±	5.0	< 0.0001	< 0.0001	< 0.0001
Initial BG	(mg/dl)	0.4506^†^	202	±	28	199		±	29	N/A	259	±	114	270	±	130	N/A	N/A	N/A
Final BG	(mg/dl)	< 0.0001^‡^	224	±	25	195		±	47	0.1878	451	±	151	513	±	111	0.2193	< 0.0001	< 0.0001
HbA1c	(%)	< 0.0001^†^	5.4	±	0.3	5.3		±	0.5	0.8442	8.8	±	3.1	10.2	±	2.6	0.5038	< 0.0001	< 0.0001

^a^Since the residuals in the two-way ANOVAs did not pass the homoscedasticity test and/or the normality test for all properties except initial body mass, p-values are from a one-way ANOVA if parametric assumptions are valid, Welch’s ANOVA if variance was different among the groups (^‡^), or the Kruskal–Wallis test if residuals still do not pass normality (^†^)

^b^Adjusted p-values used Holm-Šídák’s correction of pairwise comparison p-values from t-tests or Mann–Whitney tests within strain or within diet depending on whether each group passed normality test

^c^removed one outlier in which body mass was 1.5 times the interquartile range below the 25th percentile

Between 12 and 16-weeks, non-fasting blood glucose (BG) significantly depended on strain and the interaction between strain and age (Table 1). Some of the NZO10 animals had BG levels greater than 250 mg/dl, but this was not consistent. The ShiLtJ animals had circulating BG levels below this diabetic threshold (Fig. 1B). Taking the average measurements at 15-weeks and 16-weeks, BG did not significantly vary between the strains, irrespective of the assignment to a diet group (Table 2). Between 16-weeks and 37-weeks, circulating BG depended on age, strain, diet, and several interactions among these main effects (Table 1). Most of the NZO10 mice on HFD maxed out the glucometer with BG levels above 600 mg/dl starting at 22-weeks, whereas most of the ShiLtJ mice on HFD had BG levels above 250 mg/dl at 24-weeks (Fig. 1B). However, the elevated BG was not consistent for these HFD-fed ShiLtJ mice. Taking the average at 36-weeks and 37-weeks (after 21 weeks of purified diet), non-fasting BG was similar between LFD and HFD in this strain (Table 2). As an indicator of diabetes severity, glycated hemoglobin (HbA1c) was also not different between the 2 diet groups in ShiLtJ mice and below 6.5%. BG and HbA1c on the other hand were much higher in the NZO10 than in the ShiLtJ mice, but the diabetes severity of the NZO10 mice did not depend on diet as there were no significant differences in HbA1c between LFD and HFD within this strain (Table 2).

### High Fat Diet-Related Decrease in Trabecular Bone Volume Fraction of the Distal Femur Metaphysis was Independent of Diabetes

**Fig. 2:**
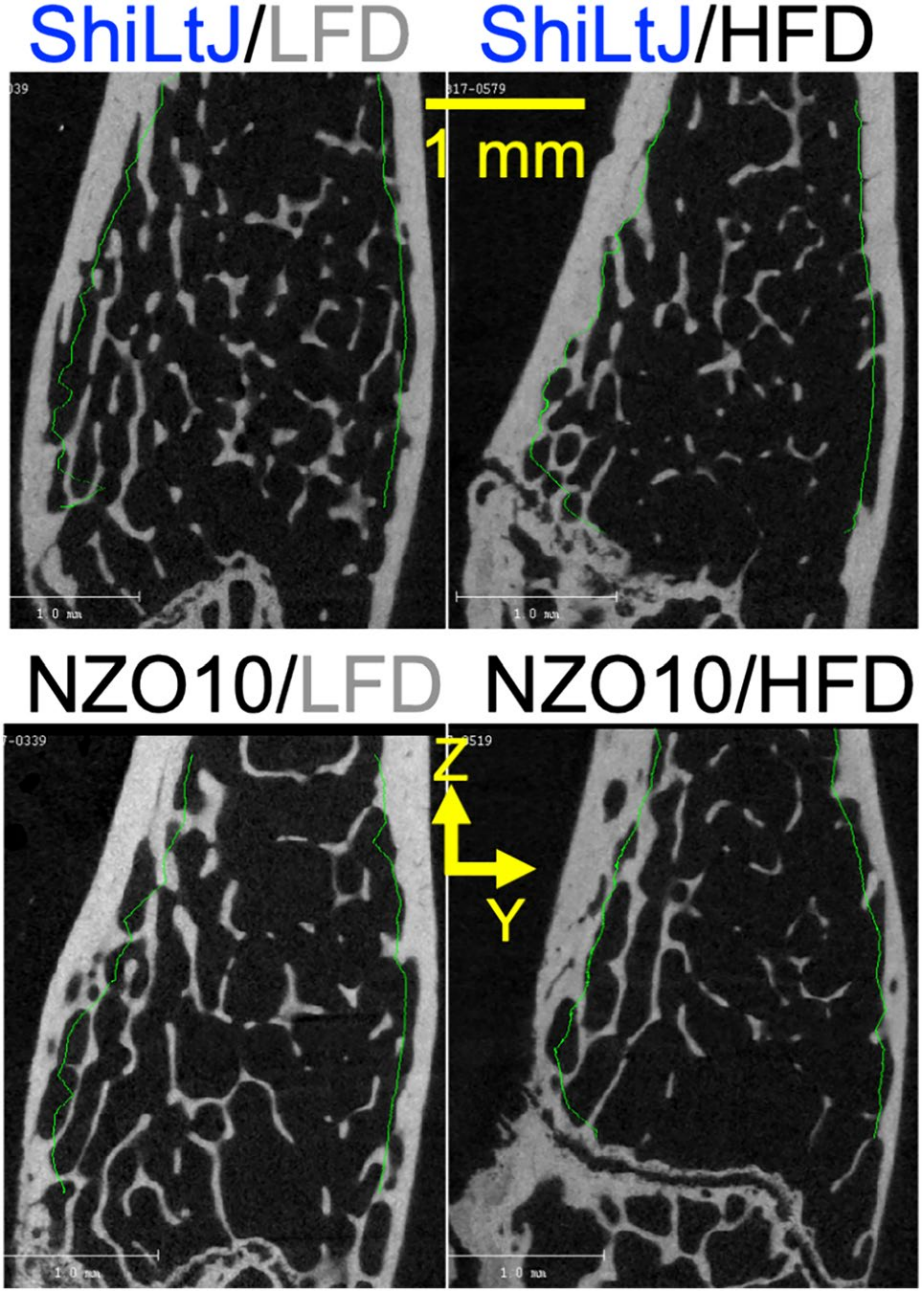
Representative μCT images of the distal femur metaphysis from each group. In longitudinal sections of the metaphysis, there appear to be fewer trabeculae in mice fed a high fat diet for 21 weeks, irrespective of strain

Diet did not affect the length of the femur, but it significantly affected trabecular bone volume fraction (BV/TV) in the distal femur metaphysis (Fig. 2) and did so independently of the strain-related T2D effect on BV/TV (Table 3). That is, the interaction between strain and diet was not significant, and BV/TV was lower in HFD than in LFD group for both ShiLtJ without diabetes and NZO10 mice with T2D. Diet, strain, and their interaction did not affect trabecular thickness (Tb.Th) nor tissue mineral density of trabecular bone (Tb.TMD). As such, the lower BV/TV in mice fed a HFD, irrespective of strain, was due to a significantly lower trabecular number (Tb.N) (Table 3). HFD increased trabecular spacing (Tb.Sp) and decreased connectivity density (Conn.D) of the trabecular bone with these parameters being significantly higher and lower, respectively, in the diabetic NZO10 mice than in non-diabetic ShiLtJ mice, irrespective of diet (Table 4).

**Table 3:** P-values from analysis of variance of properties of the femur (caliper length, μCT, RS, three-point bending, quantitative fluorescence) and tibia (HPLC)

	Two-way ANOVA			One-way
Property	Diet	Strain	Interaction	ANOVA^a^
Femur length	0.8295	< 0.0001	0.8272	
Bone volume fraction	0.0002	0.0152	0.3503	
Trabecular number				< 0.0001^‡^
Trabecular thickness	0.8952	0.1507	0.8843	
Trabecular spacing				< 0.0001^†^
Connectivity density				< 0.0001^‡^
Trabecular tissue mineral density	0.3365	0.4770	0.1928	
Bone cross-sectional area	0.3195	< 0.0001	0.6841	
Total cross-sectional area	0.4955	< 0.0001	0.6226	
Cross-sectional moment of inertia	0.6544	< 0.0001	0.5115	
Medullary volume				0.1272^‡^
Cortical thickness	0.1237	< 0.0001	0.9679	
Cortical porosity				0.0010^†^
Cortical volumetric bone mineral density	0.9341	0.5517	0.0157	
Cortical tissue mineral density	0.4777	0.1404	0.1704	
ν_1_PO_4_/CH_2_-wag				0.8981^†^
CO_3_/ν_1_PO_4_				0.0046^†^
1/FWHM[ν_1_PO_4_]	0.4205	0.1168	0.0007	
Yield moment				< 0.0001^†^
Ultimate moment				< 0.0001^†^
Post-yield displacement*	0.4259	0.0342	0.4259	
Work-to-fracture*				0.0745^‡^
Fracture toughness K_c_				< 0.0001^†^
Fluorescent advanced glycation end-products				0.1587^†^
Pentosidine concentration				0.0014^‡^

*span-adjusted determination of post-yield displacement and work-to-fracture

^a^If residuals in the two-way ANOVA did not pass the homoscedasticity test and/or the normality test for a given property, p-values came from a one-way ANOVA if parametric assumptions were valid, the Welch’s ANOVA if variance was different among the groups (^‡^), or the Kruskal–Wallis test if residuals in the one-way ANOVA did not pass normality (^†^)

**Table 4:** Effect of diet and strain on trabecular morphology (μCT), cortical structure (μCT), and mechanical properties (three-point bending) of the femur

		ShiLtJ						Diet^b^	NZO10						Diet^b^	Strain	effect^b^
Property^a^	Units	LFD		vs		HFD		effect	LFD		vs		HFD		effect	w/in LFD	w/in HFD
Length	mm	14.94	±	0.21	14.97	±	0.23	N/A	14.35	±	0.23	14.35	±	0.29	N/A	< 0.0001	< 0.0001
BV/TV	%	17.2	±	2.1	14.8	±	1.8	0.0078	15.6	±	2.0	14.1	±	1.3	0.0078	0.1721	0.2093
Tb.N	1/mm	5.04	±	0.34	4.65	±	0.44	0.0418	4.29	±	0.28	4.11	±	0.20	0.0418	< 0.0001	0.0013
Tb.Th	μm	48.3	±	3.0	48.1	±	4.2	N/A	47.0	±	1.8	47.0	±	2.7	N/A	N/A	N/A
Tb.Sp	μm	0.188	±	0.015	0.208	±	0.024	0.0402	0.229	±	0.016	0.239	±	0.012	0.0444	< 0.0001	0.0087
Conn.D	mm^−3^	234	±	31	200	±	34	0.0284	146	±	24	131	±	13	0.0339	< 0.0001	< 0.0001
Tb.TMD	mgHA/cm^3^	1067	±	14	1066	±	9	N/A	1065	±	16	1073	±	14	N/A	N/A	N/A
Ct.Ar	mm^2^	1.14	±	0.06	1.13	±	0.07	N/A	0.92	±	0.05	0.90	±	0.05	N/A	< 0.0001	< 0.0001
Tt.Ar	mm^2^	1.77	±	0.10	1.80	±	0.07	N/A	1.53	±	0.08	1.53	±	0.11	N/A	< 0.0001	< 0.0001
I_min_	mm^4^	0.167	±	0.017	0.172	±	0.017	N/A	0.113	±	0.013	0.112	±	0.015	N/A	< 0.0001	< 0.0001
Ma.V	mm^3^	1.16	±	0.12	1.23	±	0.15	N/A	1.12	±	0.08	1.17	±	0.12	N/A	N/A	N/A
Ct.Th	mm	0.218	±	0.009	0.214	±	0.012	N/A	0.202	±	0.005	0.198	±	0.011	N/A	< 0.0001	0.0014
Ct.Po	%	1.93	±	0.31	2.44	±	0.33	0.0010	2.11	±	0.20	2.20	±	0.21	0.218	0.0750	0.0473
Ct.vBMD	mgHA/cm^3^	1331	±	7	1322	±	14	0.1256	1324	±	6	1329	±	13	0.1455	0.0187	0.1607
Ct.TMD	mgHA/cm^3^	1356	±	5	1354	±	13	N/A	1356	±	8	1362	±	13	N/A	N/A	N/A
M_y_	N mm	56.7	±	4.2	51.0	±	6.7	0.0229	45.3	±	5.3	41.5	±	5.1	0.0229	< 0.0001	0.0002
M_u_	N mm	60.5	±	5.0	61.1	±	7.1	0.8284	49.5	±	4.1	46.6	±	5.1	0.1721	< 0.0001	< 0.0001
PYD*	mm^−1^	0.012	±	0.007	0.012	±	0.007	N/A	0.018	±	0.006	0.015	±	0.008	N/A	0.0922	0.3360
W_f_*	N	1.75	±	0.67	1.67	±	0.36	0.6996	1.66	±	0.25	1.38	±	0.37	0.0351	0.6567	0.0917
K_c,ult_	MPa√m	5.47	±	0.72	5.34	±	0.81	0.7393	4.18	±	0.41	3.79	±	0.33	0.0104	< 0.0001	< 0.0001

^a^Following the length of the left femur are μCT parameters of trabecular (Tb) bone and cortical (Ct) bone using standard nomenclature. Below the μCT parameters are the properties from three-point bending tests: yield moment (M_y_), ultimate moment (M_u_), post-yield displacement (PYD) adjusted for span (12 × d / span^2^)*, work-to-fracture (W_f_) using the moment vs. span-adjusted displacement* curve, and critical stress intensity factor of notched femur (K_c_)

^b^Adjusted p-values used Holm-Šídák’s correction of pairwise comparison p-values from t-tests or Mann–Whitney tests within strain or within diet depending on whether each group passed normality test

In linear regression models that include body mass (BM) as covariate, BV/TV, Tb.N, and Conn.D were still significantly lower in the HFD group than in LFD group (Table S2). The significant strain-related differences in trabecular bone (Table 4) persisted when controlling for the BM of the mice with Tb.N and Conn.D being higher in ShiLtJ than in NZO10 mice (Table S2). None of the μCT-derived architectural parameters of trabecular bone in the distal femur metaphysis (DFM) depended on BM other than possibly BV/TV (p = 0.063 for β coefficient of BM). Thus, the negative effect of HFD on trabecular bone volume architecture is independent of the weight gain that HFD caused in each strain.

### Mouse Strain, but not Diet, Affected Cortical Structure of the Femur Mid-Diaphysis.

Unlike trabecular bone of the distal femur metaphysis, HFD did not affect the structure of cortical bone (femur mid-diaphysis) as determined by μCT evaluations (Table 3). On the other hand, strain affected multiple μCT parameters with no interactions with diet (Table 3). Within both diet groups, the NZO10 mice with diabetes and lower body mass had significantly lower cross-sectional bone area (Ct.Ar), total area (Tt.Ar), and minimum moment of inertia (I_min_) than did the ShiLtJ mice (Table 3). With no significant differences in medullary volume (Ma.V) among the 4 groups (Table 3), the reduction in Ct.Ar was due to the cortices of the mid-diaphysis (Ct.Th) being thinner in the NZO10 mice than in the ShiLtJ mice (Table 3). Diet nor strain affected cortical TMD (Ct.TMD), but cortical porosity (Ct.Po) was 26% higher, on average, in ShiLtJ mice fed HFD compared to ShiLtJ mice fed LFD (Table 4). This apparent effect of HFD on micro-structure did not occur in the NZO10 mice.

Since the HFD-related gain in BM was more pronounced in the ShiLtJ than in the NZO10 mice (Fig. 1A), we also determined if strain and diet affected the μCT parameters of mid-diaphysis in linear regression models with BM as a covariate. Strain still significantly affected Ct.Ar, Tt.Ar, I_min_, section modulus (*SM* = I_min_/c_min_), though BM was only a significant contributor to Ct.Ar (Table S2). These structural parameters were significantly higher in ShiLtJ than in NZO10 mice. With BM as a covariate, diet, but not strain, significantly affected Ct.Th, and Ct.Po such that they were lower and higher in HFD- than in LFD-fed mice, respectively (Table S2). Like Ct.Ar, Ct.Th was positively related to BM. In other words, higher body mass conferred greater cortical thickness, but the equivalent BM-related increase in Ct.Th (+ 1 μm/g) in both diet groups was 3 μm lower, on average, in HFD than in LFD.

### HFD Reduced Bending Strength of the Femur Mid-Diaphysis, Irrespective of Strain, but Only Lowered Fracture Toughness in the Diabetic NZO10 Strain.

In both strains of mice, HFD lowered the yield moment that the femur mid-diaphysis endured during three-point bending (Table 4). Interestingly, there were no significant differences in the ultimate moment between the 2 diet groups within each strain (Table 4). Not surprisingly, these structural-dependent measurements of bending strength were lower in the diabetic NZO10 than in the control ShiLtJ mice given how strain affected bone structure (Table 3 and Table 4). As an indicator of possible diet-related effects on material strength of cortical bone, we regressed yield moment (M_y_) and ultimate moment (M_u_) versus section modulus (Fig. 2). For the same SM, which is a cross-sectional characteristic of a beam that directly influences bending strength, yield moment (yield force x span / 4) was, on average, lower in mice fed HFD than in mice fed LFD (Fig. 2). Diet however did not significantly affect the linear relationship between M_u_ and SM. HFD still significantly lowered M_y_ when controlling for strain and BM (Table S2).

Strain, but not diet, significantly affected span-adjusted post-yield displacement (PYD*), but in the pair-wise comparisons between strains with diet groups, the higher PYD* in NZO10 than in ShiLtJ was not significant (Table 4). A two-way ANOVA was not possible for span-adjusted work-to-fracture (W_f_*) of the intact femurs as well as fracture toughness (K_c_) of the contralateral notched femurs using the ultimate force during crack propagation (Table 3). Since the one-way ANOVA and Kruskal–Wallis test, respectively, were suggestive of significant differences among the 4 groups, we determined the adjusted p-values for pairwise comparisons of LFD vs. HFD within mouse strain and ShiLtJ vs. NZO10 within diet. In contrast to other properties of trabecular and cortical bone, HFD significantly lowered W_f_* and K_c_ in only the diabetic NZO10 mice (Table 4). K_c_, but not W_f_*, was significantly lower in NZO10 mice than in ShiLtJ mice (﻿Table 4).

**Fig. 3:**
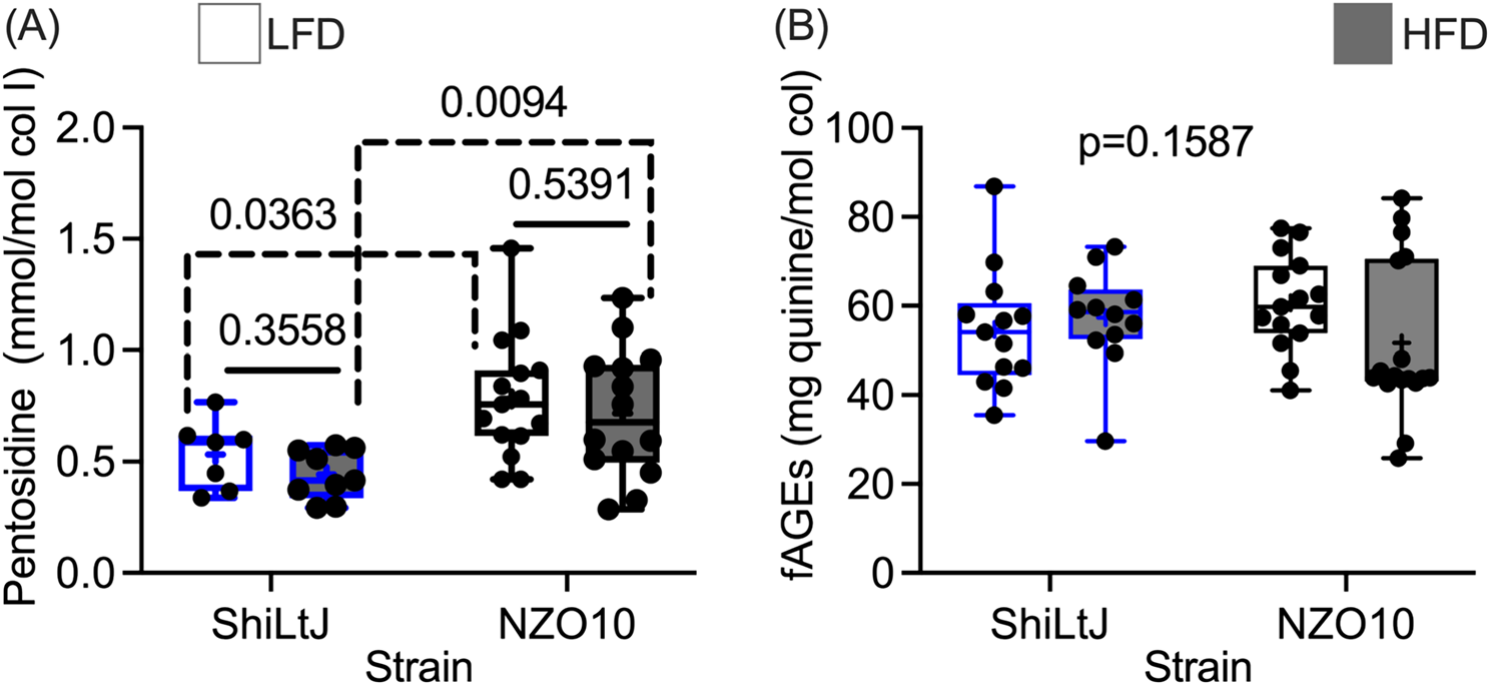
Effect of diet and strain on advanced glycation end-products (AGEs). **A** The concentration of the AGE crosslink pentosidine was higher in the diabetic NZO10 mice than in the control ShiLtJ mice. **B** However, there were no significant differences in AGEs among the 4 groups when measured by fluorescence

One possible explanation for the lower fracture toughness in the diabetic NZO10 mice is an increase in advanced glycation products (AGEs). Fluorescent AGEs (fAGEs) in notched femur were not significantly different among the 4 groups (Table 3 and Fig. 3A)). However, the concentration of the AGE crosslink called pentosidine in the tibia was significantly higher in diabetic mice compared to control mice, but HFD had no effect on this AGE (Fig. 3B). We also analyzed the compositional characteristics of the femur by Raman spectroscopy (RS). The peaks at 1150 cm^−1^ and 1345 cm^−1^ putatively considered markers of carboxy-methyl-lysine (CML) and pentosidine, respectively, were quite weak in the acquired spectra (Fig. S2). As such, we did not detect statistically significant differences in the peaks among the 4 groups (Table 3). Out of most RS properties (Table 3 and Table S4), there were only significant differences in type B carbonate substitution (CO_3_/ν_1_PO_4_) and crystallinity (inverse of full-width at half maximum of the ν_1_PO_4_ peak) among the 4 groups. In pairwise comparisons, only crystallinity was significantly higher in ShiLtJ mice fed HFD than in ShiLtJ mice fed LFD (Table S5).

### Although HFD did not Significantly Decrease BV/TV in the L6 Vertebral Body (VB), it did Reduce the Ultimate Force of the VB in Compression.

**Fig. 4:**
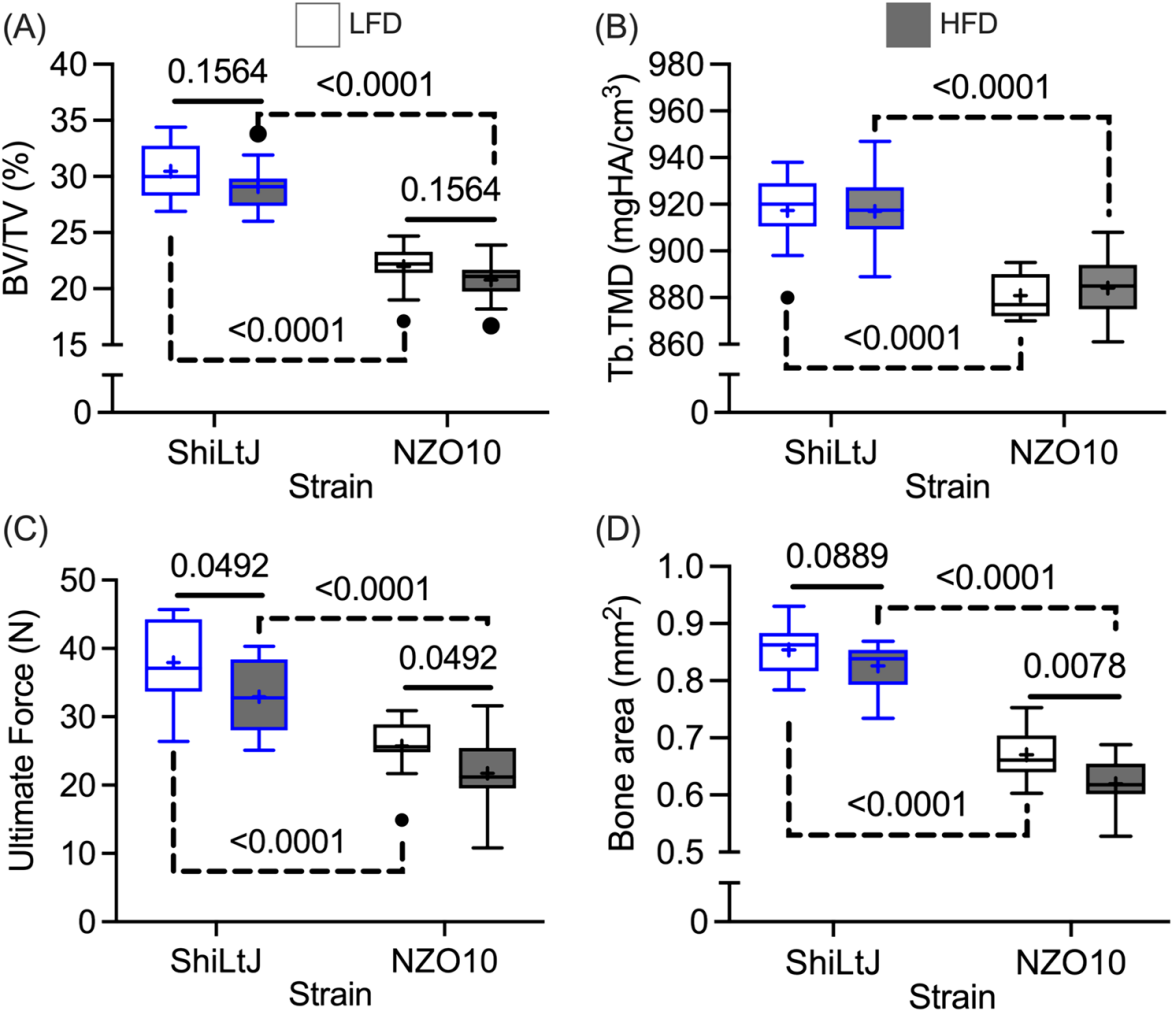
Strain- and diet-related differences in the L6 vertebral body (VB). **A** As determined by μCT evaluations, the trabecular bone fraction was lower in the NZO10 mice than in the ShiLtJ mice but wasn’t affected by diet. **B** This was also the case for tissue mineral density of the trabecular bone in the centrum. **C** Despite the lack of diet effect on trabecular bone, HFD lowered the compressive strength of the VB with this mechanical property being lower in NZO10 strain than the control strain. **D** This effect on strength could be due to low cross-sectional area of the VB in mice fed a HFD. Tukey’s outliers, 1.5 × the interquartile range, are included in the statistical analysis

The HFD-related differences in the distal femur metaphysis (Table 4) were not prominent in the L6 vertebral body (VB). The μCT parameters of trabecular bone depended on strain but not on diet (Table 5). Although HFD did not significantly reduce BV/TV (Fig. 4A) nor Tb.TMD (Fig. 4B), it weakened the strength of the L6 VB in compression (Fig. 4C) likely because it decreased the cross-sectional bone area of this vertebra (Fig. 4D). There were several differences in this axial bone between the 2 strains (Table S5).

**Table 5:** ANOVA p-values for selected properties of L6 Vertebral body

	Two-way ANOVA			One-way
Property	Diet	Strain	Inter	ANOVA
BV/TV				< 0.0001*
Tb.N	0.6411	< 0.0001	0.2637	
Tb.Th	0.0953	< 0.0001	0.6333	
Tb.Sp	0.6311	< 0.0001	0.1489	
Conn.D	0.9730	< 0.0001	0.6429	
Tb.TMD	0.6909	< 0.0001	0.6126	
Bone area	0.0013	< 0.0001	0.3476	
Yield force				< 0.0001 *
Ultimate force	0.0023	< 0.0001	0.7240	

*Residuals in the two-way ANOVA did not pass the test for heteroscedasticity

### Differences in Many Bone Properties Existed Between the 2 Strains Before NZO10 Developed Frank T2D.

To ascertain whether T2D-related differences in bone properties were independent of strain, we compared bone properties between ShiLtJ (*n* = 6) and NZO10 (*n* = 8) mice at 15 weeks of age before the mice became overtly diabetic. Similar to strain-related trends in the mice before being switched to LFD or HFD (Table 2), there was not a significant difference in body mass (*p* = 0.8778) and non-fasting glucose levels tended to be higher in NZO10 than in ShiLtJ (*p* = 0.0593, Table S7). Even though NZO10 mice had just started to become diabetic (glucose levels > 250 mg/dl) while ShiLtJ mice had not (glucose levels < 250 mg/dl), numerous differences in trabecular architecture already existed. Tb.N and Conn.D before LFD or HFD were lower in NZO10 than in ShiLtJ, while BV/TV was significantly different between the strains (Table S7). This was the case for 37-week-old mice assigned to either the LFD or HFD group (Table 4). Strain-related differences in the femur mid-diaphysis also existed at 15-weeks (Table S7) and at 37-weeks (Table 4) within each diet group. The differences were more pronounced in the older, diabetic mice (percent differences in Table S7). The cortical bone of NZO10 mice was also weaker in bending than the cortical bone of ShiLtJ at 15-weeks (Table S7).

## Discussion

There are no clear guidelines on how to treat or manage patients with type 2 diabetes or obesity such that their likelihood of suffering a fragility fracture is reduced [44]. This is a major clinical problem because diabetes and obesity increase complications following fracture fixation [45, 46]. In general, obesity favors high aBMD [47], but recent meta-analyses indicate that obesity is a risk factor for a fragility fracture [48, 49]. Identifying either T2D patients, irrespective of BMI, or obese patients, without diabetes, who are at imminent risk of a fragility fracture is rather difficult because of the dearth in knowledge about how the interactions among diet, obesity, and insulin insensitivity affect bone. By feeding mice resistant and prone to diabetes a purified diet high in fat (45% kcal) or the same purified diet with less fat (10% kcal), we found that a high level of fat, namely lard, weakens bone, irrespective of the animal’s diabetic status (normal glycated hemoglobin A1c < 6% vs. high HbA1c > 8%). This occurred in both a trabecular rich site (L6 vertebra) and a cortical site (femur mid-diaphysis). With respect to the latter, the deleterious effect of HFD on the bending strength of cortical bone was independent of the differences in body mass among the 4 different groups (Fig. 1A and Table S3). Thus, consuming a diet with high fat appears to directly lower trabecular bone volume fraction (BV/TV), particularly in long bones, and reduces the ability of cortical bone to resist yielding (i.e., the onset of permanent damage) in mice that become obese (body mass > 60 g) without diabetes and in mice that do not become obese (body mass < 45 g) but develop T2D.

The effect of HFD on trabecular architecture was less pronounced in the L6 vertebral body (VB) than in the distal femur metaphysis. A HFD-related decrease in BV/TV of lumbar VB has been observed in male, adolescent and adult C57BL/6 mice [14, 50, 51] (see #6, #9, and #17 in Table S1 for sub-strains, diets, and age range), but such an effect was not observed for male C3H/HeJ mice [52] nor female C57BL/6 mice [19] (see #18 and #24 in Table S1 for sub-strains, diets, and age range). Regardless, HFD can lower the compressive strength of a lumbar VB without significantly affecting BV/TV (Fig. 4). A non-significant decrease in BV/TV within the centrum likely combined with a non-significant (ShiLtJ) or significant (NZO10) decrease in the cross-sectional bone area of the VB to decrease in VB compressive strength in either strain of mice fed HFD (i.e., independent of T2D).

**Fig. 5:**
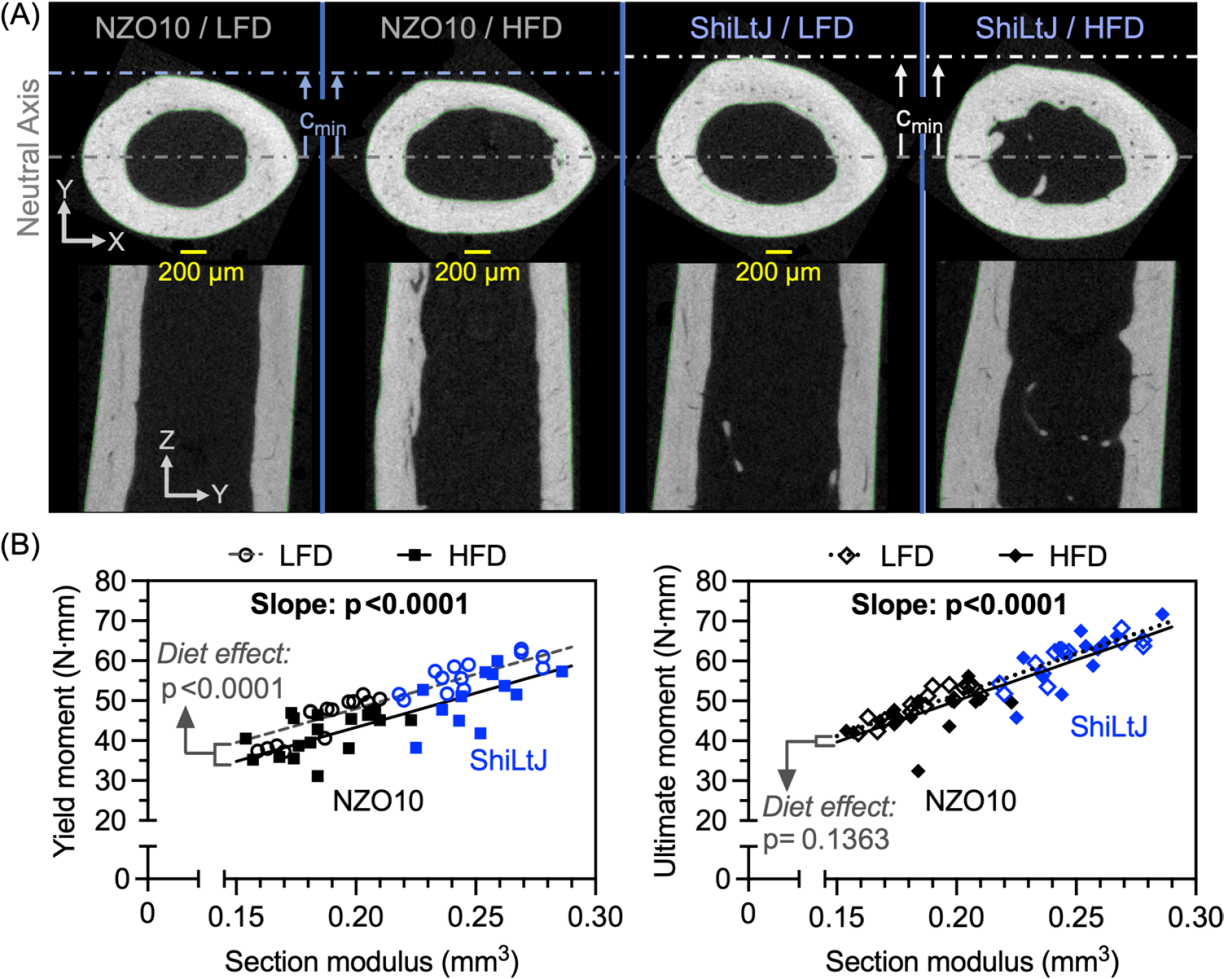
Structure and bending strength of femur mid-diaphysis. **A** In representative μCT cross-sectional images, the distance between the neutral axis (i.e., centroid) and the bone surface in direction of loading in three-point bending tests (i.e., c_min_) appears greater in the ShiLtJ control mice than the NZO10 diabetic mice. Less apparent is the smaller cortical thickness in NZO10 mice compared ShiLtJ mice as seen representative μCT longitudinal images. **B** In linear regression analysis of yield moment of femur mid-diaphysis vs. section modulus (I_min_/c_min_), HFD significantly lowers the regression line such that the cortical bone is weaker in bending for a given section modulus (left). This was not the case for ultimate moment in bending of the femur mid-diaphysis (right)

The effect of HFD on bending strength of cortical bone was not due to the high fat in the purified diet (Research Diets, Inc. D12451) causing a deterioration in the structure of the femur mid-diaphysis (FMS) since yield moment of the FMS was lower in the HFD group than in the LFD group for a given section modulus (Fig. 5B). Moreover, HFD did not significantly affect the multiple structural characteristics of the FMS when controlling for strain in an ANOVA (Tables 3, 4), thereby suggesting HFD affects the bone matrix or cortical micro-structure. Diet alone however did not significantly affect any of the selected compositional characteristics of cortical bone (Table S5). Ct.Po of the FMS was significantly higher in HFD than in LFD mice but only for the ShiLtJ strain (Table 4). Limited by the resolution of the μCT scans, Ct.Po does quantify defects in micro-structure of cortical bone that could lower the resistance of cortical bone to yielding. Although the present study did not identify the cause of the HFD-related decrease in bending strength of cortical bone, it does indicate that HFD deleteriously affects the fracture resistance in a way that cannot be simply explained by a decrease in cortical bone mass. This is congruent with clinical observations that the higher-than-expected elevation in fracture risk with T2D and obesity is independent of aBMD [5, 53].

The association between T2D and fracture risk and between obesity and fracture risk is complex. On the one hand, there is a positive correlation between BMI and aBMD unless obesity-related diseases lower bone mineral density [54–56]. On the other hand, there is a negative correlation between bone marrow adiposity, which often increases with obesity in addition to aging [57], and aBMD [58–60]. Moreover, both obese individuals [61] and those with T2D [62] are more likely to fall due to multiple factors (for risk factors for falls in obesity or T2D, see [61, 63]). In a retrospective study of 11,664 women with and without diabetes and 1:1 matching by age between non-obese (BMI < 30) and obese groups (BMI ≥ 30), non-obese women with diabetes had a high risk of vertebral fracture or hip fracture while obese women without diabetes were only at risk of non-vertebral, non-hip fractures despite higher aBMD than non-obese women [64]. There is not an equivalent study in men, but in a cross-sectional study of obese men with and without T2D, estimated failure load by finite element analysis was significantly lower in men with T2D than in men without T2D [65]. In general, T2D increases the likelihood of a fracture at any site [64], while obesity increases the likelihood of a fracture of the lower limb and humerus [66]. Although a mouse model of diet-induced obesity cannot replicate all the factors that contribute to obesity- and T2D-related increase in fracture risk, it is amendable to mechanistic studies of the role of bone marrow adiposity, AGE accumulation, and inflammation in the fracture resistance of bone.

Interestingly, HFD did not affect the ultimate moment, irrespective of mouse strain or susceptibility to diabetes, and it did not affect this measure of bending strength of bone per section modulus or structural resistance of the mid-diaphysis to bending (Fig. 5). Therefore, the HFD-related changes making bone more susceptible to the onset of permanent damage (i.e., yielding) either did not affect the maximum load carrying capacity of the cortical bone or was offset by other changes that maintain this capacity. Again, the present study was not exhaustive enough in the characterization in bone properties to provide an explanation for the discrepancy between how HFD affects yield strength and how it affected ultimate strength of cortical bone.

Another interesting observation was that HFD lowered the fracture toughness of cortical bone, namely crack initiation toughness (K_c,ult_) in only the diabetic NZO10 mice. Such an effect was observed in a previous study (#9 in Table S1) that fed growing, male C57BL/6 mice a purified diet with high fat content (60% kcal) from 4-weeks to 23-weeks of age [12]. Compared to age-, sex- and strain-matched mice fed standard chow (#3 in Table S1), the HFD-fed mice had significantly higher blood glucose only after 21-weeks of age (i.e., close to euthanasia). In a follow up study by the same group, growing mice and older mice near skeletal maturity (#7 in Table S1) were fed the same HFD, but the control mice were fed a similar purified diet with less fat (both from ResearchDiets, Inc.), not ‘chow’ [13]. This study did not report the effect of diet on K_c,ult_ (K_c_ when a crack begins to propagate at ultimate force) but rather on crack instability toughness (i.e., K_c,instability_ when the crack growth transitions from stable to instable propagation). HFD only reduced this measure of fracture toughness in the adult mice. Unlike the present study, fluorescent advanced glycation end-products (fAGEs measured in demineralized tibia) were significantly higher in only the adult mice fed HFD, while circulating glucose was significantly higher at euthanasia in both the growing and adult mice fed HFD compared to age-/sex-matched C57BL/6 mice fed LFD. There is a possibility that a longer duration of T2D (e.g., 52 weeks instead of 21 weeks) would cause the effect of diet on bone strength to be dependent on whether the mice were diabetic (i.e., significant interaction between diet and diabetes).

AGE accumulation in bone tissue as a mechanism by which HFD or diabetes reduces fracture toughness is supported by another study in which male C57BL/6 mice were fed LFD or HFD (TestDiets #58Y2 vs. TestDiets #58V8) from 10 to 32 weeks of age (#35 in Table S1). On the other hand, in a previous study by the same group in which the LFD- and HFD-fed mice were assigned to a standard 12-h of light followed by 12-h of dark cycle (12-L:12-D) per week or an altered light cycle (4 consecutive days of 12-L:12-D and then 3 consecutive days of 12-D: 12-L) per week, HFD did not significantly affect fAGEs but *increased* K_c,ult_ in both light:dark cycle groups (#32 in Table S1). Using different strains of mice, we found that HFD significantly decreased K_c,ult_ in only the diabetic NZO10 mice (confirmed by HbA1c unlike previous studies) but did not affect fAGEs. Pentosidine concentration, one specific fAGE crosslink, in bone tissue was significantly higher while K_c,ult_ was significantly lower in NZO10 than in ShiLtJ mice, regardless of diet. This association of course is not mechanistic evidence that AGE accumulation lowers the ability of cortical bone to resist crack propagation, but it does speak to the possibility that HFD and T2D can have independent effects of bone’s fracture resistance.

The strain-related differences in bone properties cannot solely be attributed to uncontrolled diabetes because ShiLtJ and NZO10 mice are not littermates but rather related. Like we observed for the diabetic TallyHO mice [39], which uses the SWR/J mouse strain as a control, and the ZDSD diabetic rat [67], which uses CD(SD) rat as a control, differences in bone properties were detected in the present study prior to the rodents becoming overtly hyperglycemic (Table S7). They may have pre-diabetes, but many of the strain-related differences (or lack thereof) in trabecular architecture of the metaphysis and in the structure of the mid-diaphysis that existed at 37-weeks of age (~ 21 weeks of T2D; Fig. 1B) also existed at 15-weeks of age. There were notable differences in the strain effect between the age groups: i) Ct.Th and K_c,ult_ of FMS as well as Tb.N, Tb.TMD, and yield force of L6 VB were not significantly different between the strains at 15-weeks but were significantly lower in the diabetic NZO10 mice than in non-diabetic ShiLtJ mice at 37-weeks of age and ii) Tb.TMD of DFM as well as Ct.TMD and span-adjusted post-yield displacement of FMS were significantly higher in NZO10 at 15-weeks but not significantly different between the strains at 37-weeks. Expressed as percent difference from ShiLtJ mice, strain-related differences in bone properties were also more pronounced with aging (i.e., progression of T2D) in both diet groups (Table S7). Therefore, T2D in NZO10 mice could be affecting bone independent of diet, but unlike diet-induced obesity (DIO), the reduced fracture resistance of the NZO10 mice was partly due to low bone mass (Table 4).

In addition to not exhaustively investigating characteristic bone matrix, several other limitations of the study include: (i) testing effects of diet and diabetes in only male mice, (ii) lack of serum markers of metabolism, (iii) an imbalance in the sample size between ShiLtJ and NZO10 mice, and (iv) treatment of mice with a glucose lowering drug. One, the DIO model has been primarily investigated in male mice (Table S1) because the metabolic disturbances caused by the HFD are more pronounced in male than in female mice. Likewise, most mouse models of T2D involve male mice because the penetrance of the diabetes phenotype is more prevalent in males than in females (see review by [9]). Knowing whether HFD affects the fracture resistance of bone in female mice with or without the onset of T2D would be useful since obesity is not sex-specific in humans and fragility fractures are more common in women than men. Two, characterizing serum and plasma markers (resorption CTX, formation P1NP, sclerostin, insulin, insulin-like growth factor 1, IL-6, TNFα, etc.) would identify potential metabolic pathways by which diet and diabetes lower the fracture resistance of bone. Three, there being more NZO10 mice per diet than ShiLtJ mice per diet possibly favored the detection of diet-related differences in the diabetic mice, but only K_c,ult_ and bone area of the VB were found to be significantly different between LFD and HFD in NZO10 mice but not in ShiLtJ mice. Four, T2D rarely goes untreated, and without treating ShiLtJ and NZO10 on LFD with a glucose-lower agent, the present did not further investigate the effects of hyperglycemia on the fracture resistance of bone. The challenge with this approach is the possibility that the selected agent has direct effects on body mass or bone, though treating ShiLtJ mice would be sensitive to such effects assuming the mice do not become hypoglycemic.

The present study was not designed to test a mechanism by which obesity or diabetes lowers the fracture resistance of bone. However, the present model of diet-induced obesity with and without T2D could be used to investigate whether the HFD-related decline in bone volume, mass, and strength is due to obesity and/or T2D inducing (i) inflammation that favors osteoclastic activity and bone resorption, (ii) expansion of marrow adipocytes (fat storage) at the expense of osteoblastic activity and bone formation, and/or (iii) increase in AGE receptor (RAGE) signaling that favors bone resorption over bone formation.

## Conclusion

The consumption of diet high in fat lowers the strength of both cortical and trabecular bone, regardless of whether the mice develop overt type 2 diabetes. In the diabetic mice, HFD also lowered the fracture toughness of cortical bone without affecting advanced glycation end-products. The overall HFD-related decrease in the fracture resistance of cortical bone was not explained by a decline in bone mass (i.e., structural resistance to bending). Feeding mice resistant to diabetes and mice prone to diabetes, irrespective of diet, a high- and low-fat diet provides a model to investigate different mechanisms by obesity without diabetes and diabetes without obesity affect the fracture resistance of bone.

### Supplementary Information

Below is the link to the electronic supplementary material.

Supplementary file1 (DOCX 2009 KB)
