# Supplementary material for: Bone Fragility in High Fat Diet-induced Obesity is Partially Independent of Type 2 Diabetes in Mice
Source: Calcif Tissue Int. 2024 Jul 16;115(3):298–314. doi: 10.1007/s00223-024-01252-x (PMC11333511; doi:10.1007/s00223-024-01252-x)
Supplement: Supplementary file 1 — Supplementary file1 (DOCX 2009 KB) [file 223_2024_1252_MOESM1_ESM.docx]

**High Fat Diet-Related Decrease in the Fracture Resistance of Mouse Bone is Partially Independent of Type 2 Diabetes**

Sasidhar Uppuganti, Amy Creecy, Daniel Fernandes, Kate Garrett, Kara Donovan, Rafay Ahmed, Paul Voziyan, Elizabeth Rendina-Ruedy, and Jeffry S. Nyman

| **Table of Contents** | **Page #** |
| --- | --- |
| **Table S1**: Summary of High Fat Diet-induced Obesity (DIO) studies reporting effects on body mass, glucose metabolism, bone structure, and bone’s resistance to fracture. (* study included mechanical testing of bone ex vivo). | 2 |
| **Table S2:** Summary of the µCT scan parameters, regions of interest (ROIs), and evaluation settings for bone segmentation of the femurs and 6^th^ lumbar vertebra | 12 |
| **Table S3**: Estimated coefficients, p-values from general linear regression models to determine if mouse strain and diet affected selected bone properties while including body mass of the animal. | 13 |
| **Table S4:** P-values from analysis of variance of Raman properties of the femur mid-diaphysis not reported in Table 3. | 13 |
| **Table S5:** Effect of diet and strain on composition (RS) of the femur mid-diaphysis | 14 |
| **Table S6:** Effect of diet and strain on micro-architectural and mechanical properties of the L6 vertebral body not reported in Fig. 5. | 14 |
| **Table S7:** Effect of strain at 15-weeks of age, before mice were fed low fat diet (LFD) or high fat diet (HFD), on bone properties, bodyweight, and non-fasting blood glucose. | 15 |
| **Figure S1**. Representative μCT images of the micro-notch on the anterior side of the femur mid-diaphysis. | 17 |
| **Figure S2**. Analysis of bone composition by Raman spectroscopy with background subtraction and filtering depicted on the left and locations of peak intensity or integrated band regions (inset) on the right. | 18 |
| **Figure S3**. Fracture toughness testing of mouse femur mid-diaphysis in which a micro-notch was introduced prior to loading in three-point bending. | 18 |
| **References** for Table S1. | 19 |

**Table S1**: Summary of High Fat Diet-induced Obesity (DIO) studies reporting effects on body mass, glucose metabolism, bone structure, and bone’s resistance to fracture. (* study included mechanical testing of bone ex vivo).

| **Strain** | **Diet** | **Age range of diet (weeks)** | **Body mass (g)**  **mean ± SD (% gain)** | **Circulating glucose (mg/dl)**  **mean ± SD** | **Effect of HFD relative to control diet** | **Ref** |
| --- | --- | --- | --- | --- | --- | --- |
| Male C57BL/6J  (n=7/diet) | NIH-31 Rodent Diet 7013 (6% fat) vs. Harland Teklad TD90221 (1.25% cholesterol, 15.8% fat, and 0.5% cholate) | 4 to 36 | Not reported | Not reported | Decrease in femoral and L4 VB mineral content | [1] |
| Male C3H/HeJ  (n=7/diet) |  |  |  |  | No significant difference in femoral and L4 VB bone mineral content |  |
| Male C57BL/6N  (n=10-11/diet) | Research Diets D12450 (10 kcal% fat, 70 kcal% carb, 20 kcal% protein) vs. Research Diets HFD (45 kcal% fat, 35 kcal% carb, 20 kcal% protein) | 6 to 20 | 31 ± 1 vs. 41 ± 2 (31% heavier & stable) | Not reported | Decrease in trabecular BV/TV (PTM), but no significant differences in Ct.Th and Ct.Ar (TMS) | [2] |
| Male C57Bl/6  (n=14-15/diet) | PicoLab mouse diet (21.6 kcal% fat, 55.2 kcal% carb., 23.2 kcal% prot.) vs. Research Diets HFD (60 kcal% fat, 20 kcal% carb., 20 kcal% prot.) | 4 to 23 | 34.7 ± 2.1 vs. 47.3 ± 3.4 (36% heavier & stable) | Hyperglycemia in the last week only; 160 vs. 220 (S at 22-wks) *non-fasting* | Decrease in yield and ultimate stress (FMS); Decrease in crack initiation toughness (FMS); Increase in cortical thickness (FMS) | [3] * |
| Female C57BL/6J  (n=15-17/diet) | Harlan Teklad LM-485 vs. 10% corn oil with AIN93 composition | 52 to 78 | (39% heavier still rising) | 86.0 ± 3.97 vs. 178.5 ± 5.2 (S at 78-wks)  *6-8 hour fasting* | Decrease in aBMD (DFM / PTM) and Decrease in aBMD (FMS / TMS) | [4] |
| Male wild-type and apoE^−/−^ mice on the C57BL/6J | Control Diet (Bio-Serv F4031, 7wt.% fat) vs. Diabetogenic HFD (Bio-Serv F3282, 35 wt.% fat) | 4 to 20 | (Wild-type 35% heavier & rising and apoE^-/-^ 20% heavier & stable) | wild-type 140 ± 25 vs. 175 ± 25 and apoE^-/-^ 120 ± 25 vs. 150 ± 25 | Decrease in BV/TV, Tb.N, and Tb.Th (non-significant) In apoE^-/-^ group. No significant differences in wild-type group. | [5] |
| Male C57BL/6J  (n=6/diet) | Research Diets low fat control diet (10% of total calories from lipids) vs. Research Diets HFD (60% from lipids) | 7 to 31 | (52.1% heavier) | Not reported but insulin plasma levels were 8.6-fold higher | Decrease in whole-body aBMD and decrease in BV/TV (L4 VB) | [6] |
| Male C57BL/6  (n=14-15/diet) | Research Diets LFD (10 kcal% fat, 70 kcal% carb., 20 kcal% protein) vs. Research Diets HFD (60 kcal% fat, 20 kcal% carb., 20 kcal% protein) | 3 to 19 | (44.2% heavier & stable) | 97.7 ± 16.3 vs. 187.7 ± 39.1 (S at 19-wks)  *overnight fasting* | Decrease in yield and ultimate stress (FMS); No difference in crack instability toughness (FMS); No difference in cortical thickness (FMS) | [7] * |
|  |  | 15 to 31 | (52.7% heavier stable) | 99.4 ± 29.8 vs. 191.9 ± 41.1 (S at 31-wks)  *overnight fasting* | Decrease in ultimate stress only (FMS); Decrease in crack instability toughness (FMS); Decrease in cortical thickness (FMS) |  |
| Male C57BL/6J  (n=8/group) | Oriental Yeast Co. standard diet (16.4% fat per total cal) vs. HFD (62.2% fat per total cal) | 7 to 11, 15, or 19 | 27.8±1.2 vs. 32.6±1.7 (S at 11-wks) 31.8±2.3 vs. 41.5±2.3 (S at 15-wks) and 33.7±3.3 vs. 45.3±2.5 (S at 19-wks) | Not reported | Decrease in BV/TV and Tb.N (PTM) starting at 11-wks. Decrease in cortical bone density at 19-wks and Decrease in Ct.Ar at 15- and 19-wks. (TMS) | [8] |
| Male C57Bl/6J  (n=7-8/diet/age) | ResearchDiets LFD (10 kcal% fat) vs. Research Diets HFD (60 kcal% fat) | 5 to 17 | (37.5% heavier) | ~150 vs. ~300 *overnight fasting* | Decrease in trabecular BV/TV (DFM and L3 VB) *Decrease in ultimate compressive force (L3 VB)* | [9] * |
|  |  | 20 to 32 | (78.5% heavier) | ~100 vs. ~340 *overnight fasting* | Decrease in trabecular BV/TV (DFM and L3 VB)  Decrease in ultimate compressive force (VB) |  |
| Male C57Bl/6  (n=10/diet) | Standard AIN-93G diet (14% fat) vs. HFD (45% fat, 25% protein, and 30% carbohydrate at 375 kcal/kg) | [23-24 g] for 8 weeks | (15.8% heavier) | Not reported | No significant difference in trabecular vBMD (PTM); Decrease in trabecular vBMD (PTM) if normalized by body mass; Decrease in ultimate force (FMS) | [10] * |
| Male C57BL/6  (n=10/diet) | Control diet (10% kcal fat) vs. Research Diets D12492a HFD (60% kcal from fat) | 6 to 18 | 32.9 ± 4.2 g vs. 43.6 ± 4.0 g (S ~32.5% heavier) | Not reported | Decrease in trabecular vBMD, BV/TV, and Conn.D (DFM). Increase in periosteal circumference and Decrease in Ct.Ar and Ct.Th (FMS) | [11] |
| Male C57BL/6J  (n=6-7/diet) | Altromin (Lage, Germany) normal diet (10% kcal from fat) vs. Altromin HFD (60% kcal from fat) | 4 to 23 | (11% higher at 27-wks of age) relative stable | Not reported | No difference in whole body aBMD. Decrease in bone area by histology (DFM) | [12] |
| Female C57BL/6  (n=5/diet/group) | PicoLab Mouse low fat Diet 20 (5058) vs. Research Diets 12541 (45% fat, 35% carbohydrate and 20% protein) | 8 to 14 | 7% lighter in exercise group, 11% heavier in non-exercise group at 6 weeks | Not reported | No significant differences in BV/TV, Tb.N, Tb.Th, Tb.Sp, Ct.Ar, Tt.Ar, and Ct.Th between HFD and control mice. | [13] |
| Male C57BL/6J  (n=4-6/diet) | Teklad Global 2016 (12 kcal% fat) vs. ResearchDiets D12451 (45 kcal% fat) | 12 to 23 | (31% heavier) | Not reported but GTT AUC was 60% higher | Increase in trabecular BV/TV (PTM) and cortical section modulus and in Ct.Th (TMS) but a decrease in BFR (PTM) | [14] |
| Male C57Bl/6J mice  (n=10/diet) | Research Diets D12450B diet (10% kcal from fat) vs. Research Diets D12492 HFD (60% kcal from fat) | 3 to 15 | (37% heavier stable) | Not reported | NS decrease in BV/TV. Decrease in Tb.N and Conn.D (DFM). No difference in Ct.Ar, Tt.Ar, Ct.Th, and Ct.Po (FMS) | [15] |
| Male C57BL/6J  (n=4-6/diet/age) | Research Diets D12450B LFD (10 kcal% from fat) vs. Research Diets D12492 HFD (60 kcal% from fat) | 5 to 8, 11, or 17 | (42% heavier) at 17-wks. | 76 vs. 100 (31.5% higher) at 11-wks. and 78 vs. 124 (59.0% higher) at 17-wks. *fasting* | Decrease in BV/TV, Tb.N, and Tb.Th (DFM) at 11- and 17-wks. of age (6 and 12 weeks of HFD) | [16] |
| Male C57BL/6J  (n=15/diet) | AIN-93M (10 kcal% from fat) vs. Harlan Teklad TD.06415 (45 kcal% from fat) | 9 to 33 | (27.2% heavier) | 150 vs. 200 and GTT AUC was 44% higher | Decrease in whole-body BMC but no differences in vBMD and BV/TV (L4). No difference in cortical thickness (TMS) | [17] |
| Male C57BL/6N  (n=15/diet) |  |  | (40.9% heavier) | 150 vs. 200 and GTT AUC was 44% higher | Decrease in whole-body BMC and decrease in vBMD and BV/TV (L4) but no significant difference in Ct.Th (TMS) |  |
| Male C3H/HeJ  (n=15/diet) |  |  | (28.9% heavier) | 150 vs. 150 and GTT AUC was 36% higher | Decrease in whole-body BMC but no significant differences in vBMD and BV/TV (L4). No difference in Ct.Th (TMS) |  |
| Male C57BL/6N  (n=16/group) | AIN-93M (10% kcals from fat) vs. Research Diets D12492 HFD (60 % kcals from fat) | 4 to 6, 12, or 20 | (8.3%, 35.0%, and 69.5% heavier at 6-, 12-, and 20-wks, respectively) | 156.1 ± 28.8 vs. 185.9 ± 22.0, 151.1 ± 21.6 vs. 182.3 ± 29.2, and 147.9 ± 8.4 vs. 178.2 ± 24.4 (S at all ages)  *6-hour fasting* | Decrease in aBMD (whole-body) and BV/TV (DFM) at 12- and 20-wks. No difference in BV/TV (L4 VB). Decrease in Ct.Th and Ct.Ar (FMS) at 12-wks only. Increase in 1^st^ ID (DFM) at 12- and 20-wks. Increase in 1^st^ ID (FMS) at 20-wks. | [18] |
| Male C3H/HeJ  (n=16/group) |  |  | (17.7%, 35.6%, and 43.5% heavier at 6-, 12-, and 20-wks, respectively) | 163.8 ± 17.6 vs. 195.3 ± 24.8, 132.5 ± 19.2 vs. 161.3 ± 23.6, and 129.3 ± 13.6 vs. 134.9 ± 24.0 (S at 6- and 12-wks)  *6-hour fasting* | Decrease in aBMD (whole-body) at 20-wks. No difference in BV/TV (L4 VB and DFM). Increase in Ct.Th (FMS) at 20-wks only. Decrease in 1^st^ ID (FMS) at 6-wks., but an Increase in 1^st^ ID (FMS) at 20-wks. |  |
| Male C57BL/6N  (n=8-10/group) | Modified AIN-93M Research Diets D12450J (sucrose matched, 10% kcals from fat) vs. Research Diets D12492 (60% kcals from fat) | 5 to 7, 13, and 21 | Not reported | Elevated fasting blood glucose (175 mg/dL HFD vs. 150 mg/dL control), impaired 120 min GTT (600 mg/dL HFD vs. 200 mg/dL control), elevated plasma insulin (736.7 pg/mL HFD vs. 370.8 pg/mL control) at 16 weeks | Decrease in BV/TV, Tb.N (non-significant), and Tb.Th. Increase in Tb.Sp (non-significant) at 16 weeks. | [19] |
| Male NIH Swiss  mice (n=8/diet) | Standardized rodent chow (10% fat, 30% protein, 60% carbohydrate; percent of total energy 12.99 kJ/g; vs. HFD (45% fat, 20% protein, 35% carbohydrate; percent of total energy 26.15 kJ/g) | 8 to 32 | (36.3% heavier) | [4.9 ± 0.5 mM vs. 22.5 ± 7.5 mM]  *Non-fasting*; GTT AUC was 45% | No significant differences in ultimate force (FMS) but a decrease in ultimate displacement such that work-to-fracture was lower (FMS) | [20] * |
| Male C57BL/6 mice  (n=8/diet) | Standard chow diet group vs. Research Diets D12109C HFD (40% kcal fat) | 4 to 16 | (25% heavier) | Not reported | Decrease in BV/TV, Tb.Th, Tb.N (PTM) at 12 weeks; Decreases in ultimate force and in work-to-fracture (FMS). | [21] |
| Male C57BL/6N mice  (n=10/diet/age) | Standard rodent chow diet (AIN76A, 5% fat from corn oil, 11.5% fat) vs. HFD (20% fat from corn oil, 37.8% fat) | 3 to 22, 52, or 78 | 45 ± 4 vs. 51 ± 8 at 78-wks | Not reported | Decrease in vBMD, BV/TV, and Tb.N (DFM) at 22-, 52-, and 78-wks. No difference in Tb.Th (DFM). Decrease in cortical vBMD, Ct.TMD, Ct.Ar, and Ct.Th (FMS) at 22-, 52-, and 78-wks. Decrease in yield force at 22-wks. but Increase in ultimate force at 52- and 78-wks (FMS). | [22] * |
| Male C57Bl/6J mice  (n=7-8/diet/age) | Normal chow diet (ND, 13.5% calories from fat) vs. Research Diets D12492 HFD (60% calories from fat) vs weight loss group (WL, HFD for 12 weeks then ND for 8 weeks) | 6 to 12, 16, or 20 | (50%, 72%, 79% heavier at 12-, 16-, and 20-wks, respectively) | Not reported | Decrease in BV/TV, BMC, and Tb.N at 12-, 16-, and 20-wks. (PTM). No difference in Tb.Th (PTM). Weight loss partially rescued decreases in trabecular BV/TV, BMC, and Tb.N. No significant difference in cortical morphology (TMS), but Decrease in Ct.Th, which was rescued by weight loss, at 20-wks. (FMS). At 20-wks, decrease in yield force, ultimate force, and post-yield work (not PYD). | [23] * |
| Female C57BL/6J  (n=10/diet/age) | Research Diets 12450B, (10 kcal% fat, 20 kcal% protein, 70 kcal% carbohydrate) vs.  Research Diets 12451 (45 kcal% fat, 20 kcal% protein, 35 kcal% carbohydrate) | 3 to 6, 12, or 20 | (22% heavier still rising) | Not reported but impaired GTT | Decreases in BV/TV and in Tb.N (DFM; S at 12-wks and S at 12-wks and 20-wks, respectively); No difference in BV/TV but Decreases in Tb.N and in Conn.D (L5 VB; S at 6-wks and 12-wks and S at 6-, 12-, and 20-wks); Increase in pMOI (FMS; significant at 12- and 20-wks); No difference in Ct.Th, ultimate force, and PYD (FMS) | [24] * |
| Female FVB/NJ  (n=10/diet/age) |  |  | (No difference still rising) | Not reported; less impaired GTT | Decreases in BV/TV and in Tb.N (DFM; S at 6-wks for both properties); No differences in BV/TV, in Tb.N, and in Conn.D (L5 VB); Decrease in pMOI (FMS; S at 12-wks only); Decrease in (FMS; S at 20-wks only); No difference in Ct.Th and in PYD (FMS) |  |
| Male C57BL/6 mice  (n=14/diet/age) | Normal chow diet (12% fat, 64% carbohydrate, 24% protein) vs HFD (60% fat, 20% carbohydrate, 20% protein) | 6 to 12 | (14% heavier) | 35% higher glucose and 112% higher plasma insulin  *fasting* | Decrease in BV/TV and Tb.Th but no difference in Tb.N (Histology of PTM). Decrease in BV/TV, Tb.N, Ct.Th, and Ct.vBMD (μCT of DFM and FMS); Decreases in ultimate force per bone area and in work-to-fracture (FMS). | [25] * |
| C57BL/6 mice  (n=10/diet) |  | 6-8 to 18-20 |  |  |  | [26] * |
| Female LG/J and male SM/J inter-cross strain  (n=274/LFD/sex, n=283/HFD/sex) | Low fat chow (LF, 15% calories from fat) vs. high fat chow (HF, 42% calories from fat) | 21 to 29 | 25.0 ± 4.1 (LF, female vs. 33.8 ± 9.40 (HF, female). and 35.1 ± 5.3 (LF, male) vs 45.7 ± 7.4 (HF, male) | Not reported | Increases in Ct.Ar and Ct.Th (FMS), but no difference in pMOI. Increases in ultimate force and work-to-fracture; No difference in post-yield displacement. Mechanical properties positively correlated with body mass. | [27] * |
| Male NIH Swiss mice  (n=8/diet) | Normal chow (10% fat, 30% protein, 60% carbohydrate) vs. HFD (45% fat, 20% protein, 35% carbohydrate) | 8 to 21 (150 days) | 45 ± 2 vs. 56 ± 1 (24% heavier) | 417 ± 32 vs. 614 ± 40 glucose tolerance (mmol/l/min). 97 ± 11 (NC) vs. 58 ± 10 (HFD) insulin sensitivity (ng/ml/min). | No difference in whole body, lumbar, or femoral aBMD. Decrease in ultimate force, post-yield displacement, and work-to-fracture. Decrease in ultimate stress. Decreases in indentation modulus and hardness. Decreases in BV/TV, Tb.N, Tt.Ar, Ct.Ar, and Ct.Th (PTM and TMS) | [28] * |
| Male *Dkk1^fl/fl^;Rosa26‐CreER^T2^* and *Dkk1^fl/fl^;Osx‐Cre*  (n=20-26/diet) | Normal diet (9% fat, 58% carbohydrates, 33% protein) vs. HFD (60% fat, 20% carbohydrate, 20% protein) | 8 to 20 | (16-19% increase vs. 40-45% increase  *Stable*) | Not reported but decreased GTT | Decrease in BV/TV and Tb.N (DFM) for cre- (WT) and cre+ (cKO). Decrease in Ct.Th and BMD (FMS) for WT only | [29] |
| Male C57BL/6N mice  (n=12-13/diet) |  |  |  |  |  | [30] |
| Male C57Bl/6J (n=7/diet/age) | Normal chow (NC) SF09-091 diet (16.1 MJ/kg) vs. SF02-006 HFD (24 MJ/kg with 60% fat) | 8 to 18 | 29 ± 1.2 vs. 26.3 ± 0.5 | Not reported but impaired GTT | Decrease in BV/TV (PTM); Decrease in Ct.Th (TMS); Decrease in ultimate force (FMS) | [31] * |
|  |  | 12 to 22 | 29 ± 0.5 vs. 31.4 ± 0.9 | Not reported but impaired GTT | No significant difference in BV/TV (PTM); Decrease in Ct.Th (TMS); Decrease in ultimate force (FMS) |  |
| Male C57/BL6  (n=12/diet/sleep cycle) | Low fat diet (10% fat, 72% carbs, 18% proteins) vs. high fat diet (46% fat, 36% carbs, 18% proteins) | 10 to 32 | (20% heavier) | 15% higher  *fasting* | Decrease in Ct.Th for normal light and altered light cycle (FMS); Increase in vBMD for normal but decrease in vBMD for altered light cycle (FMS); No difference in moment of inertia, regardless of light cycle (FMS). No difference in vBMD and BV/TV, but a decrease in Tb.N, namely in the altered light cycle (PTM). Increase in yield stress combining both light cycle groups, but diet had opposite effects on ultimate stress between light cycle groups (TMS). Increase in fracture toughness in both light:dark cycle groups, but diet didn’t affect AGEs with AGEs being higher in altered light. | [32] * |
| Male C57BL/6J  (n=5/diet) | Ctrl diet (20% kcal protein, 70% kcal carbohydrate, 10% kcal fat) vs. high poly-unsaturated fat (PUFA: 20% kcal protein, 20% kcal carbohydrate, 60% kcal fat) vs. high saturated fat (HSF: 20% kcal protein, 20% kcal carbohydrate, 60% kcal fat) vs. 50:50 mix of saturated: unsaturated fatty acids (HFD 50/50: 20% kcal protein, 20% kcal carbohydrate, 60%kcal fat) | 10 to 18 | 27.02 ± 0.25 (control) vs. 41.22 ± 1.42 (PUFA) vs. vs. 36.24 ± 0.59 (HSF) vs. 39.66 ± 2.1 (HFD 50/50) (still rising with control diet cause less gain in body mass | Not reported | The different diets of saturated to unsaturated did not have a profound effect on cortical and trabecular bone with saturated fat being more problematic than unsaturated fat. Diets high in omega-9 appeared to be better than diets low in this fatty acid. | [33] * |
| Male, Institute of Cancer Research (ICR) mice  (n=9/diet) | Normal control (NC) vs. Research Diets 12032 HFD (45% fat) | NR to 12 and 22 | 46.4 ± 1.8 vs. 54.4 ± 7.4 (17.2% heavier at 12^th^ week) and 44.0 ± 2.8 vs. 66.2 ± 7.3 (33% heavier at 22^nd^ week) | 5.35 ± 0.98 vs. 8.13 ± 0.86 mM/L (NS at 12^th^ week) and 5.92 ± 0.80 vs. 9.06 ± 1.01 mM/L (S at 22^nd^ week) *fasting BG* | Decrease in BV/TV, BS/TV, Tb.N, Tb.Th, and Conn.D, but an increase in TMD and Tb.Sp (PTM). Decrease in TMD, Tr.Ar, and Ct.Th but an increase in Ct.Ar and Ma.Ar (TMS). Decrease in ultimate force per area (TMS). | [34] * |
| Male C57Bl/6J  (n=6/diet) | TestDiets #58Y2 LFD (10% fat, 72% carbs, 18% proteins) vs. TestDiets #58V8 HFD (46% fat, 36% carbs, 18% proteins) | 10 to 32 | (30.5% heavier) | NS difference at 16-weeks (200 vs. 210); S difference at 23-weeks (200 vs. 230) and at 31-weeks (220 vs. 290) | Increase in BV/TV and vBMD (DFM); No significant difference in Ct.Th (FMS); *Increase in fAGEs (Femur)*; Decrease in crack initiation toughness (FMS) | [35] * |
| Male BALB/c  (n=5/group) | Research Diets D12450B control diet (10% of calories from fat) vs. Research Diets D12451 HFD (45% of calories from fat) | 6 to 26 | (HFD-Obese > 20% heavier than LFD; HFD-Non-Obese ≤ 5% heavier than LFD & stable) | Not reported | Decrease in BV/TV and Tb.N (PTM and DFM) but no differences in these properties between HFD-Non-Obese and LFD; Decrease in TMD (TMS), ultimate stress and ultimate force (FMS) between LFD and obese HFD; No significant differences in Ct.Th (TMS) among the 3 groups | [36] * |
| Male C57Bl/6J  (n=8/diet) | Low fat diet (10 % kcal fat as soybean oil and lard) vs. high fat diet (46 % kcal fat as soybean oil and lard) vs. high fat diet supplemented with ground English walnut (HF + walnut; 46 % kcal fat as soybean oil, lard, and walnut) | 9 to 17 | 46.5% heavier in HF + walnut vs control, 29% heavier in HF vs control | Not reported | No significant differences in cortical volume, marrow volume, cortical thickness, or polar moment of inertia between groups (FMS). Decrease in bone volume fraction and connectivity density in HF vs. LF (DFM). No significant difference in Tb.Th, Tb.N, or Tb.Sp. No significant differences in BV/TV, Conn.D, Tb.Th, Tb.N, or Tb.Sp between groups for distal femur epiphysis. No significant differences in femur BMD between groups. | [37] |
| Female and Male wild-type and preptin knock-out (KO) mice  (n=11-15 / diet / sex / genotype) | SF16-074 LFD (14.0% kcal from fat) vs. SF04-027 HFD containing (46.0% kcal from fat) | 9 to 23 | Female WT (6.0% heavier & stable) | NS difference (7.0 vs. 7.8 mmol/L) *fasting BG* | Decrease in BV/TV, Tb.Th, and Tb.N (DFM) in male and female mice, irrespective of genotype  Decrease in Ct.Ar, Ct.Th, pMOI, and fracture load (FMS) in male and female mice, irrespective of genotype | [38] |
|  |  |  | Female KO (6.3% heavier & stable) | NS difference (7.5 vs. 7.8 mmol/L) *fasting BG* |  |  |
|  |  |  | Male WT (8.6% heavier & stable) | NS difference (9.2 vs. 9.6 mmol/L) *fasting BG* |  |  |
|  |  |  | Male KO (9.2% heavier & stable) | NS difference (7.5 vs. 9.7 mmol/L) *fasting BG* |  |  |

Low fat diet (LFD), High fat diet (HFD), Not significant (NS), Significant (S), Proximal tibia metaphysis (PTM), Tibia mid-shaft (TMS), Distal femur metaphysis (DFM), Femur mid-shaft (FMS), lumbar (L) vertebral body (VB) Glucose tolerance test (GTT), Trabecular bone volume fraction (BV/TV), Trabecular number (Tb.N), Trabecular thickness (Tb.Th),Trabecular spacing (Tb.Sp), Connectivity density (Conn.D), Cortical thickness (Ct.Th), Cross-sectional cortical bone area (Ct.Ar), Cross-sectional polar moment of inertia of mid-diaphysis (pMOI)

**Table S2:** Summary of the µCT scan parameters, regions of interest (ROIs), and evaluation settings for bone segmentation of the femurs and 6^th^ lumbar vertebra

| **Settings** | **Units** | **Left femur** | | **Right femur** ^a^ | **L6 vertebra** |
| --- | --- | --- | --- | --- | --- |
| *Scan parameters* |  |  | |  |  |
| Sample tube holder dimensions | mm (Ø) x mm (L) | 6.0 x 45.0 | | 9.0 x 78.0 | 9.0 x 78.0 |
| Peak X-ray voltage | kVp | 70.0 | | 70.0 | 55.0 |
| X-ray tube current | µA | 114.0 | | 114.0 | 200.0 |
| X-ray power | W | 8.0 | | 8.0 | 11.0 |
| Beam filter size | mm | 0.1 Al | | 0.5 Al | 0.5 Al |
| Field-of-view (F.O.V) | mm | 7.0 | | 7.0 | 10.2 |
| Integration time | ms | 600 | | 600 | 600 |
| Data average | No. | 1 | | 1 | 2 |
| Samples acquired | No. | 1024 | | 1024 | 1024 |
| Projections per 180° rotation | No. | 500 | | 500 | 500 |
| Voxel size | µm | 6.0 | | 6.0 | 12.0 |
| Axial slices of scan | No. | FMD ^b^ | DFM ^c^ | 310 | 240 |
|  |  | 310 | 620 |  |  |
| *ROI selection* |  | FMD | DFM |  |  |
| Slice count in z-stack | n | 310 | 450 | 230±13 | 173±14 |
| Image matrix size | pixels | 1162 x 1162 x 310 | | 1162 x 1162 x 310 | 494 x 494 x 240 |
| *Image segmentation* |  | FMD | DFM |  |  |
| Gaussian sigma |  | 0.2 | 0.2 | 0.8 | 0.2 |
| Gaussian Support |  | 1.0 | 1.0 | 1.0 | 1.0 |
| Lower threshold | mgHA/cm^3^ | 912.6 | 441.5 | 926.3 | 583.9 |
| Upper threshold | mgHA/cm^3^ | 2229.3 | 2229.3 | 2787.3 | 2787.3 |

^a^ micro-notched; ^b^ Femur mid-diaphysis (FMD); ^c^ Distal femur metaphysis (DFM)

**Table S3**: Estimated coefficients, p-values from general linear regression models to determine if mouse strain and diet affected selected bone properties while including body mass of the animal.

| **Property** | **Ref ^a^** (NZO10) | **Strain ^b^** (ShiLtJ) | **Diet** (HFD) **^c^** | **Body mass ^d^** | **Units** | **Adj-R^2^** |
| --- | --- | --- | --- | --- | --- | --- |
| BV/TV = | + 12.3 | p=0.814 | -2.7, p<0.0001 | x +0.09, p=0.063 | (%) | 0.304 |
| Tb.N = | + 3.91 | +0.52, p=0.0002 | -0.37, p=0.002 | p=0.199 | (1/mm) | 0.561 |
| Tb.Th = | + 43.3 | p=0.983 | p=0.364 | p=0.219 | (μm) | 0.014 |
| Tb.Sp = | + 243.7 | -31.1 p<0.0001 | +18.4, p=0.003 | p=0.330 | (μm) | 0.578 |
| Conn.D = | + 124.6 | +70.6, p<0.0001 | -29.4, p=0.003 | p=0.343 | (mm^-3^) | 0.716 |
| Tb.TMD = | +1061 | p=0.448 | p=568 | p=0.694 | (mgHA/cm^3^) | -0.18 |
| Ct.Ar = | + 0.708 | + 0.163, p<0.0001 | -0.065, p=0.001 | x +0.005, p=0.005 | (mm^2^) | 0.821 |
| Tt.Ar = | + 1.458 | + 0.241, p<0.0001 | p=0.838 | p=0.692 | (mm^2^) | 0.643 |
| I_min_ = | + 0.091 | + 0.051, p<0.0001 | p=0.569 | p=0.205 | (mm^4^) | 0.772 |
| SM = | + 0.155 | + 0.054, p<0.0001 | p=0.313 | p=0.101 | (mm^3^) | 0.755 |
| Ct.Th = | + 0.165 | p=0.179 | -0.003, p=0.0001 | x +0.001, p<0.0001 | (mm) | 0.553 |
| Ct.Po = | + 1.62 | p=0.434 | +0.23, p=0.026 | p=0.195 | (%) | 0.231 |
| Ct.TMD = | + 1346 | - 7.73, p=0.088 | p=0.990 | p=0.318 | (mgHA/cm^3^) | 0.020 |
| M_y_ = | + 37.4 | + 7.9, p=0.0005 | -6.5, p=0.001 | p=0.131 | (N mm) | 0.618 |
| M_u_ = | + 36.1 | + 9.1, p<0.0001 | -4.3, p=0.025 | x +0.32, p=0.024 | (N mm) | 0.618 |

**^a^** Reference is the β_0_ estimate for NZO10 on LFD (p<0.0001 for all properties indicating they are > 0)

**^b^** Add (+) or subtract (-) the β_1_ estimate for ShiLtJ mice to reference unless strain did not significantly affect property

**^c^** Add (+) or subtract (-) the β_2_ estimate for mice in the HFD group to reference unless diet did not significantly affect property

**^d^** Multiply β_3_ estimate by body mass of mouse and add (+) to reference unless body mass did not significantly affect property

**Table S4:** P-values from analysis of variance of Raman properties of the femur mid-diaphysis not reported in Table 3.

|  | Two-way ANOVA | | | One-way |
| --- | --- | --- | --- | --- |
| Property | Strain | Diet | Interaction | ANOVA ^a^ |
| ν_1_PO_4_/Amide I | 0.3361 | 0.2507 | 0.9922 |  |
| ν_1_PO_4_/Proline | 0.4383 | 0.1600 | 0.8805 |  |
| ν_1_PO_4_/Amide III |  |  |  | 0.7852 ^†^ |
| OH-Proline/Proline | 0.5953 | 0.5217 | 0.5463 |  |
| A_1365-1386_/ν_1_PO_4_ [GAGs] | 0.8467 | 0.3809 | 0.6527 |  |
| A_1492-1503_/CH_2_-wag [PEN] |  |  |  | 0.6647 |
| A_1147-1153_/CH_2_-wag [CML] | 0.6255 | 0.3458 | 0.2021 |  |

^a^ If residuals in the two-way ANOVA did not pass the homoscedasticity test and/or the normality test for a given property, p-values came from a one-way ANOVA if parametric assumptions were valid, the Welch’s ANOVA if variance was different among the groups (^‡^), or the Kruskal-Wallis test if residuals in the one-way ANOVA did not pass normality (^†^).

**Table S5:** Effect of diet and strain on composition (RS) of the femur mid-diaphysis.

|  |  | ShiLtJ | | | | | | Diet ^b^ | NZO10 | | | | | | Diet ^b^ | Strain | effect ^b^ |
| --- | --- | --- | --- | --- | --- | --- | --- | --- | --- | --- | --- | --- | --- | --- | --- | --- | --- |
| Property ^a^ | Units | LFD | | vs. | | HFD | | effect | LFD | | vs. | | HFD | | effect | w/in LFD | w/in HFD |
| ν_1_PO_4_/Amide I | - | 43.34 | ± | 6.66 | 45.18 | ± | 5.38 | N/A | 44.88 | ± | 6.21 | 46.69 | ± | 5.27 | N/A | N/A | N/A |
| ν_1_PO_4_/Proline | - | 39.96 | ± | 2.44 | 41.05 | ± | 3.17 | N/A | 39.52 | ± | 2.27 | 40.41 | ± | 2.55 | N/A | N/A | N/A |
| ν_1_PO_4_/Amide III | - | 15.85 | ± | 2.57 | 15.51 | ± | 4.75 | N/A | 16.11 | ± | 4.06 | 15.65 | ± | 4.19 | N/A | N/A | N/A |
| ν_1_PO_4_/CH_2_-wag | - | 13.94 | ± | 1.77 | 13.79 | ± | 3.23 | N/A | 14.28 | ± | 2.68 | 13.89 | ± | 2.39 | N/A | N/A | N/A |
| OH-Proline/Proline | - | 0.291 | ± | 0.039 | 0.304 | ± | 0.038 | N/A | 0.303 | ± | 0.030 | 0.303 | ± | 0.049 | N/A | N/A | N/A |
| CO_3_/ν_1_PO_4_ | - | 0.1386 | ± | 0.0037 | 0.1334 | ± | 0.0084 | 0.1370 | 0.1349 | ± | 0.0065 | 0.1315 | ± | 0.0039 | 0.1243 | 0.1009 | 0.4109 |
| 1/FWHM[ν_1_PO_4_] | cm | 0.0576 | ± | 0.0002 | 0.0580 | ± | 0.0004 | 0.0030 | 0.0576 | ± | 0.0003 | 0.0578 | ± | 0.0003 | 0.1551 | 0.5237 | 0.2611 |
| I_1365-1386_/ν_1_PO_4_ [GAGs] | - | 0.0216 | ± | 0.0069 | 0.0249 | ± | 0.0120 | N/A | 0.0222 | ± | 0.0101 | 0.0233 | ± | 0.0077 | N/A | N/A | N/A |
| I_1492-1503_/CH_2_-wag [PEN] | - | 0.0728 | ± | 0.0084 | 0.0786 | ± | 0.0201 | N/A | 0.0730 | ± | 0.0160 | 0.0766 | ± | 0.0126 | N/A | N/A | N/A |
| I_1147-1153_/CH_2_-wag [CML] | - | 0.0053 | ± | 0.0022 | 0.0070 | ± | 0.0036 | N/A | 0.0067 | ± | 0.0030 | 0.0064 | ± | 0.0023 | N/A | N/A | N/A |

**Table S6:** Effect of diet and strain on micro-architectural and mechanical properties of the L6 vertebral body not reported in Fig. 5.

|  |  | ShiLtJ | | | | | | Diet ^b^ | NZO10 | | | | | | Diet ^b^ | Strain | effect ^b^ |
| --- | --- | --- | --- | --- | --- | --- | --- | --- | --- | --- | --- | --- | --- | --- | --- | --- | --- |
| Property ^a^ | Units | LFD | | vs. | | HFD | | effect | LFD | | vs. | | HFD | | effect | w/in LFD | w/in HFD |
| Tb.N | 1/mm | 5.107 | ± | 0.315 | 5.155 | ± | 0.243 | N/A | 4.677 | ± | 0.267 | 4.562 | ± | 0.245 | N/A | 0.0014 | <0.0001 |
| Tb.Th | μm | 60 | ± | 2 | 58 | ± | 3 | N/A | 49 | ± | 2 | 49 | ± | 2 | N/A | <0.0001 | <0.0001 |
| Tb.Sp | μm | 187 | ± | 15 | 183 | ± | 12 | N/A | 209 | ± | 13 | 216 | ± | 13 | N/A | 0.0002 | <0.0001 |
| Conn.D | mm^-3^ | 216.74 | ± | 28.91 | 219.82 | ± | 16.58 | N/A | 185.76 | ± | 18.57 | 183.11 | ± | 25.02 | N/A | 0.0021 | <0.0001 |
| Yield force | N | 35.1 | ± | 6.3 | 31.3 | ± | 7.3 | 0.1719 | 24.5 | ± | 4.0 | 20.7 | ± | 4.9 | 0.0459 | <0.0001 | <0.0001 |

^b^ Adjusted p-values used Holm-Šídák’s correction of pairwise comparison p-values from t-tests or Mann-Whitney tests within strain or within diet depending on whether each group passed normality test.

**Table S7:** Effect of strain at 15-weeks of age, before mice were fed low fat diet (LFD) or high fat diet (HFD), on bone properties, bodyweight, and non-fasting blood glucose.

|  |  | 15-wks of age | | | | | | | | 37- | wks |
| --- | --- | --- | --- | --- | --- | --- | --- | --- | --- | --- | --- |
| Property | Units | ShiLtJ | | | cNZO10 | | | Before diet %^a^ | p-value^b^ | LFD %^c^ | HFD %^d^ |
| Body mass | (g) | 33.0 | ± | 0.6 | 32.8 | ± | 2.1 | NS^d^ | 0.8778 | -24.0 | -27.1 |
| Glucose | (mg/dl) | 226 | ± | 19 | 253 | ± | 20 | +11.9^ | 0.0593 | +101.3 | +163.1 |
| Femur length | (mm) | 14.9 | ± | 0.2 | 14.1 | ± | 0.4 | -5.4 | 0.0007 | -3.9 | -4.1 |
| *Left distal femur metaphysis* | |  |  |  |  |  |  |  |  |  |  |
| BV/TV | (%) | 20.3 | ± | 3.8 | 20.2 | ± | 3.0 | NS | 0.8518 | NS | NS |
| Tb.N | (1/mm) | 5.41 | ± | 0.35 | 4.93 | ± | 0.29 | -8.8 | 0.0200 | -17.5 | -11.6 |
| Tb.Th | (μm) | 49.2 | ± | 3.4 | 51.1 | ± | 3.2 | NS | 0.2824 | NS | NS |
| Tb.Sp | (μm) | 174 | ± | 11 | 198 | ± | 12 | +13.8 | 0.0123 | +21.8 | +14.9 |
| Conn.D | (mm^-3^) | 280.2 | ± | 24.6 | 194.9 | ± | 7.6 | -30.4 | 0.0007 | -37.6 | -34.5 |
| Tb.TMD | (mgHA/cm^3^) | 1040 | ± | 17 | 1059 | ± | 13 | +1.8 | 0.0426 | NS | NS |
| *Left femur mid-diaphysis* | |  |  |  |  |  |  |  |  |  |  |
| Ct.Ar | (mm^2^) | 0.933 | ± | 0.043 | 0.866 | ± | 0.057 | -7.2 | 0.0127 | -19.3 | -20.4 |
| Tt.Ar | (mm^2^) | 1.617 | ± | 0.034 | 1.459 | ± | 0.109 | -9.8 | 0.0127 | -13.6 | -15.0 |
| I_min_ | (mm^4^) | 0.129 | ± | 0.005 | 0.098 | ± | 0.012 | -23.9 | 0.0007 | -32.3 | -34.9 |
| Ma.V | (mm^3^) | 1.264 | ± | 0.087 | 0.954 | ± | 0.385 | -24.6 | 0.0047 | NS | NS |
| Ct.Th | (mm) | 0.177 | ± | 0.008 | 0.175 | ± | 0.008 | NS | 0.5924 | -7.3 | -7.5 |
| Ct.Po | (%) | 2.9 | ± | 0.9 | 2.4 | ± | 0.4 | NS | 0.3450 | +9.3^ | -9.8 |
| Ct.TMD | (mgHA/cm3) | 1315 | ± | 19 | 1336 | ± | 16 | +1.6 | 0.0426 | NS | NS |
| Yield moment | (N mm) | 39.6 | ± | 1.4 | 37.2 | ± | 3.3 | -6.2 | 0.0426 | -20.1 | -18.6 |
| Ultimate moment | (N mm) | 46.0 | ± | 1.0 | 41.4 | ± | 4.1 | -10.0 | 0.0200 | -18.2 | -23.7 |
| PYD* | (1/mm) | 0.0178 | ± | 0.0092 | 0.0388 | ± | 0.0169 | +117.3 | 0.0280 | +50.0^ | NS |
| Work-to-fracture* | (kJ/m^2^) | 1.46 | ± | 0.39 | 2.18 | ± | 0.64 | 49.3 | 0.0426 | NS | -17.4^ |
| *Right femur mid-diaphysis (notched)* | | |  |  |  |  |  |  |  |  |  |
| ν_1_PO_4_/Amide I |  | 39.7 | ± | 5.76 | 43.2 | ± | 5.30 | NS | 0.5728 | NS | NS |
| ν_1_PO_4_/Proline |  | 35.7 | ± | 1.78 | 35.8 | ± | 2.57 | NS | 0.7546 | NS | NS |
| ν_1_PO_4_/Amide III |  | 14.1 | ± | 2.74 | 12.3 | ± | 1.07 | NS | 0.1419 | NS | NS |
| ν_1_PO_4_/CH_2_-wag |  | 12.5 | ± | 1.89 | 11.5 | ± | 0.86 | NS | 0.4136 | NS | NS |
| OH-Proline/Proline |  | 0.298 | ± | 0.0361 | 0.330 | ± | 0.0372 | NS | 0.1419 | NS | NS |
| CO_3_/ν_1_PO_4_ |  | 0.132 | ± | 0.0025 | 0.126 | ± | 0.0039 | -4.5 | 0.0103 | NS | NS |
| 1/FWHM[ν_1_PO_4_] | (cm) | 0.0571 | ± | 0.0003 | 0.0570 | ± | 0.0002 | NS | 0.3570 | NS | NS |
| A_1365-1386_/ν_1_PO_4_ [GAGs] |  | 0.0243 | ± | 0.0068 | 0.0297 | ± | 0.0047 | 22.2^ | 0.0593 | NS | NS |
| A_1492-1503_/CH_2_-wag [PEN] |  | 0.0778 | ± | 0.0075 | 0.0813 | ± | 0.0095 | NS | 0.2824 | NS | NS |
| A_1147-1153_/CH_2_-wag [CML] |  | 0.0058 | ± | 0.0022 | 0.0072 | ± | 0.0012 | NS | 0.1812 | NS | NS |
| K_c,ult_ | (MPa√m) | 4.82 | ± | 0.74 | 4.53 | ± | 0.53 | NS | 0.3277 | -23.6 | -29.0 |
| Pentosidine |  |  |  |  |  |  |  |  |  |  |  |
| fAGEs | (mg quinine/  mol col I) | 45.7 | ± | 8.01 | 44.7 | ± | 11.7 | NS | 0.8518 | NS | NS |
| *L6 vertebral body* | |  |  |  |  |  |  |  |  |  |  |
| BV/TV | (%) | 29.2 | ± | 4.0 | 24.2 | ± | 1.9 | -16.9 | 0.0127 | -27.9 | -28.5 |
| Tb.N | (1/mm) | 4.98 | ± | 0.18 | 4.82 | ± | 0.19 | NS | 0.1812 | -8.4 | -11.5 |
| Tb.Th | (μm) | 57.3 | ± | 3.6 | 52.0 | ± | 2.6 | -9.2 | 0.0200 | -17.3 | -16.8 |
| Tb.Sp | (μm) | 196 | ± | 9 | 205 | ± | 9 | NS | 0.1419 | +12.2 | +18.0 |
| Conn.D | (mm^-3^) | 231 | ± | 14 | 217 | ± | 23 | NS | 0.2284 | -14.3 | -16.7 |
| Tb.TMD | (mgHA/cm^3^) | 863 | ± | 24 | 864 | ± | 19 | NS | 0.9497 | -4.0 | -3.6 |
| Bone Area | (mm^2^) | 0.823 | ± | 0.073 | 0.681 | ± | 0.045 | -17.3 | 0.0027 | -21.5 | -25.0 |
| Yield force | (N) | 32.4 | ± | 8.2 | 25.1 | ± | 8.9 | -22.5^ | 0.0813 | -30.2 | -33.8 |
| Ultimate force | (N) | 33.5 | ± | 7.4 | 27.5 | ± | 8.1 | NS | 0.1419 | -32.2 | -34.0 |

^a^ Percent difference was 100 x (mean of ShiLtJ – mean of NZO10) / mean of ShiLtJ at 15 weeks of age

^b^ P-values are from Mann-Whitney test comparing properties between ShiLtJ mice and NZO10 mice at 15 weeks of age.

^c^ Percent difference was 100 x (mean of ShiLtJ in LFD – mean of NZO10 in LFD) / mean of ShiLtJ in LFD at 37 weeks of age

^d^ Percent difference was 100 x (mean of ShiLtJ in HFD – mean of NZO10 in HFD) / mean of ShiLtJ in HFD at 37 weeks of age

^e^ Not significant (p>0.1)

^0.05 < p < 0.1


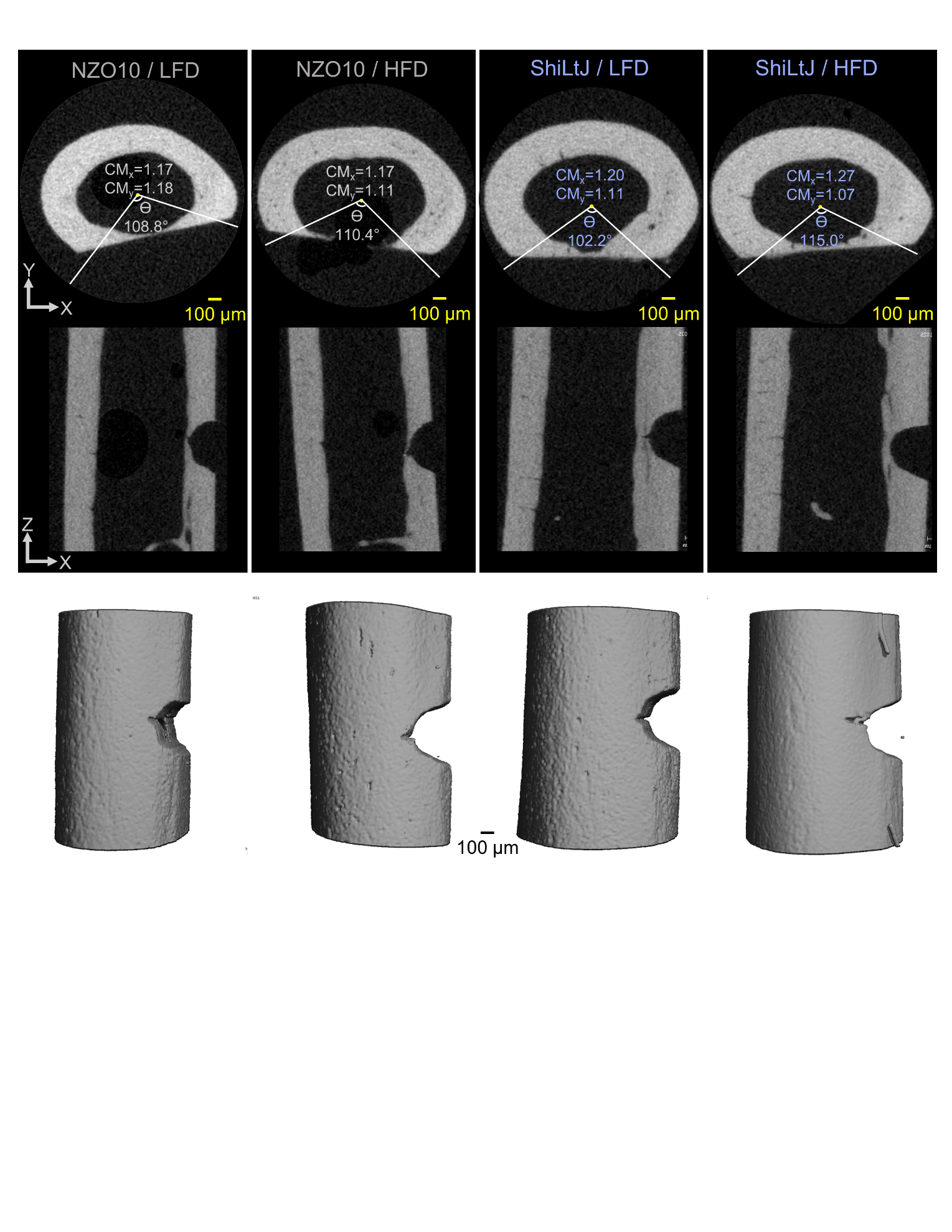


**Figure S1**. Representative μCT images of the micro-notch on the anterior side of the femur mid-diaphysis.


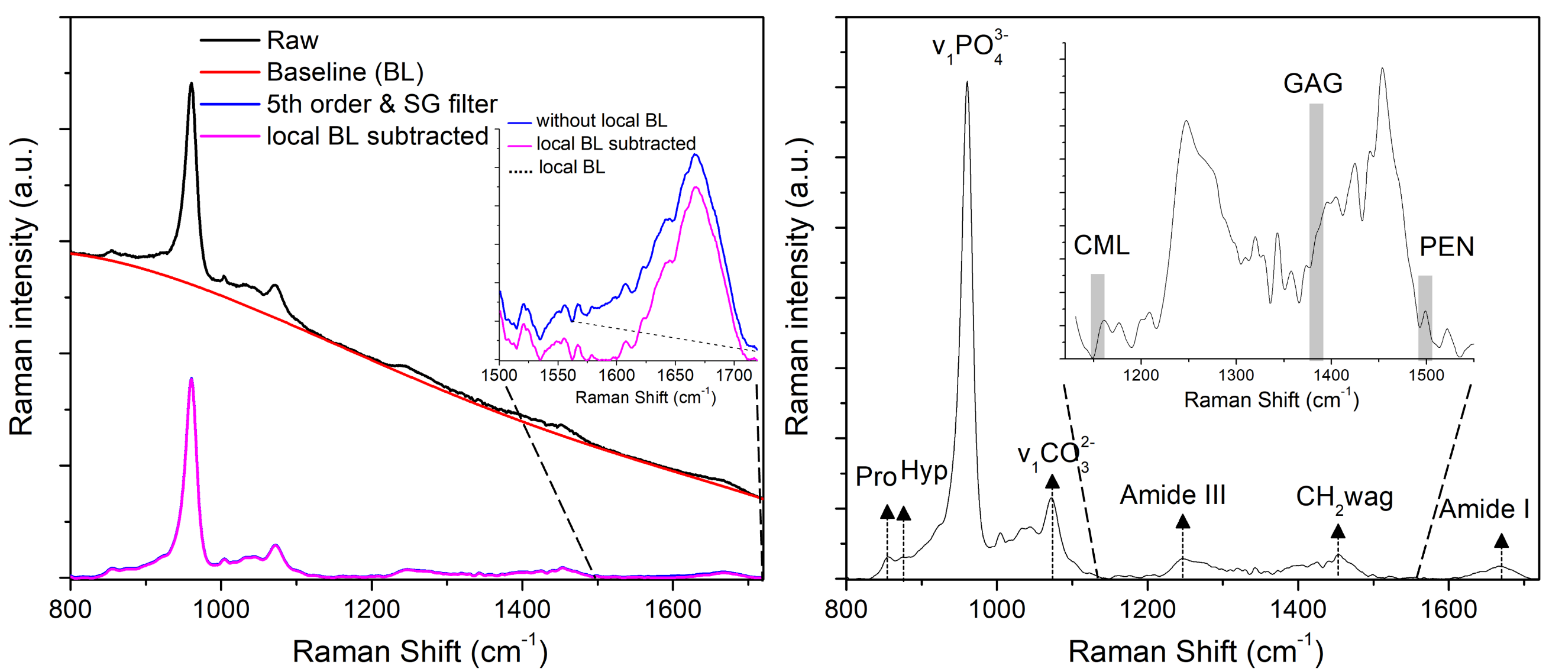


**Figure S2**. Analysis of bone composition by Raman spectroscopy with background subtraction and filtering depicted on the left and locations of peak intensity or integrated band regions (inset) on the right.


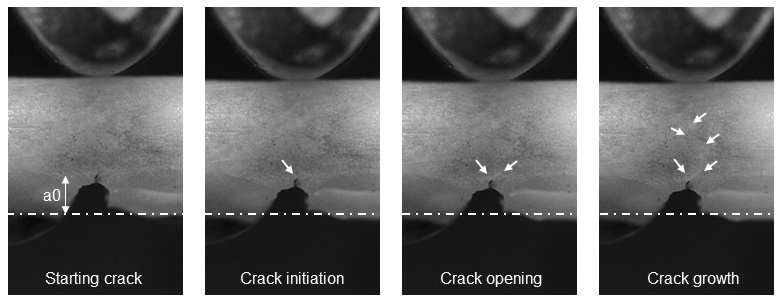


**Figure S3**. Fracture toughness testing of mouse femur mid-diaphysis in which a micro-notch was introduced prior to loading in three-point bending.

**References** for Table S1.

1. Parhami F, Tintut Y, Beamer WG, et al (2001) Atherogenic High‐Fat Diet Reduces Bone Mineralization in Mice. J Bone Miner Res 16:182–188. <https://doi.org/10.1359/jbmr.2001.16.1.182>

2. Cao JJ, Gregoire BR, Gao H (2009) High-fat diet decreases cancellous bone mass but has no effect on cortical bone mass in the tibia in mice. Bone 44:1097–1104. <https://doi.org/10.1016/j.bone.2009.02.017>

3. Ionova-Martin SS, Do SH, Barth HD, et al (2010) Reduced size-independent mechanical properties of cortical bone in high-fat diet-induced obesity. Bone 46:217–225. <https://doi.org/10.1016/j.bone.2009.10.015>

4. Halade GV, Rahman MM, Williams PJ, Fernandes G (2010) High fat diet-induced animal model of age-associated obesity and osteoporosis. J Nutritional Biochem 21:1162–1169. <https://doi.org/10.1016/j.jnutbio.2009.10.002>

5. Bartelt A, Beil FT, Schinke T, et al (2010) Apolipoprotein E-dependent inverse regulation of vertebral bone and adipose tissue mass in C57Bl/6 mice: Modulation by diet-induced obesity. Bone 47:736–745. <https://doi.org/10.1016/j.bone.2010.07.002>

6. Patsch JM, Kiefer FW, Varga P, et al (2011) Increased bone resorption and impaired bone microarchitecture in short-term and extended high-fat diet–induced obesity. Metabolis 60:243–249. <https://doi.org/10.1016/j.metabol.2009.11.023>

7. Ionova-Martin SS, Wade JM, Tang S, et al (2011) Changes in cortical bone response to high-fat diet from adolescence to adulthood in mice. Osteoporos Int 22:2283–2293. <https://doi.org/10.1007/s00198-010-1432-x>

8. Fujita Y, Watanabe K, Maki K (2012) Serum leptin levels negatively correlate with trabecular bone mineral density in high-fat diet-induced obesity mice. J Musculoskelet neuronal Interact 12:84–94

9. Inzana JA, Kung M, Shu L, et al (2013) Immature mice are more susceptible to the detrimental effects of high fat diet on cancellous bone in the distal femur. Bone 57:174–183. <https://doi.org/10.1016/j.bone.2013.08.003>

10. LU X-M, ZHAO H, WANG E-H (2013) A high-fat diet induces obesity and impairs bone acquisition in young male mice. Mol Med Rep 7:1203–1208. <https://doi.org/10.3892/mmr.2013.1297>

11. Baek K, Hwang HR, Park H, et al (2014) TNF‐α Upregulates Sclerostin Expression in Obese Mice Fed a High‐Fat Diet. J Cell Physiol 229:640–650. <https://doi.org/10.1002/jcp.24487>

12. Fehrendt H, Linn T, Hartmann S, et al (2014) Negative Influence of a Long-Term High-Fat Diet on Murine Bone Architecture. Int J Endocrinol 2014:318924. <https://doi.org/10.1155/2014/318924>

13. Styner M, Thompson WR, Galior K, et al (2014) Bone marrow fat accumulation accelerated by high fat diet is suppressed by exercise. Bone 64:39–46. <https://doi.org/10.1016/j.bone.2014.03.044>

14. Lecka-Czernik B, Stechschulte LA, Czernik PJ, Dowling AR (2015) High bone mass in adult mice with diet-induced obesity results from a combination of initial increase in bone mass followed by attenuation in bone formation; implications for high bone mass and decreased bone quality in obesity. Mol Cell Endocrinol 410:35–41. <https://doi.org/10.1016/j.mce.2015.01.001>

15. Doucette CR, Horowitz MC, Berry R, et al (2015) A High Fat Diet Increases Bone Marrow Adipose Tissue (MAT) But Does Not Alter Trabecular or Cortical Bone Mass in C57BL/6J Mice. J Cell Physiol 230:2032–2037. <https://doi.org/10.1002/jcp.24954>

16. Shu L, Beier E, Sheu T, et al (2015) High-Fat Diet Causes Bone Loss in Young Mice by Promoting Osteoclastogenesis Through Alteration of the Bone Marrow Environment. Calcified Tissue Int 96:313–323. <https://doi.org/10.1007/s00223-015-9954-z>

17. Rendina-Ruedy E, Hembree KD, Sasaki A, et al (2015) A Comparative Study of the Metabolic and Skeletal Response of C57BL/6J and C57BL/6N Mice in a Diet-Induced Model of Type 2 Diabetes. J Nutrition Metabolism 2015:758080. <https://doi.org/10.1155/2015/758080>

18. Rendina-Ruedy E, Graef JL, Davis MR, et al (2016) Strain differences in the attenuation of bone accrual in a young growing mouse model of insulin resistance. J Bone Miner Metab 34:380–394. <https://doi.org/10.1007/s00774-015-0685-z>

19. Rendina-Ruedy E, Graef JL, Lightfoot SA, et al (2016) Impaired glucose tolerance attenuates bone accrual by promoting the maturation of osteoblasts: Role of Beclin1-mediated autophagy. Bone Reports 5:199–207. <https://doi.org/10.1016/j.bonr.2016.08.001>

20. Mabilleau G, Perrot R, Flatt PR, et al (2016) High fat-fed diabetic mice present with profound alterations of the osteocyte network. Bone 90:99–106. <https://doi.org/10.1016/j.bone.2016.06.008>

21. Gautam J, Khedgikar V, Choudhary D, et al (2016) An isoflavone cladrin prevents high‐fat diet‐induced bone loss and inhibits the expression of adipogenic gene regulators in 3T3‐L1 adipocyte. J Pharm Pharmacol 68:1051–1063. <https://doi.org/10.1111/jphp.12562>

22. Aslam MN, Jepsen KJ, Khoury B, et al (2016) Bone structure and function in male C57BL/6 mice: Effects of a high-fat Western-style diet with or without trace minerals. Bone Reports 5:141–149. <https://doi.org/10.1016/j.bonr.2016.05.002>

23. Scheller EL, Khoury B, Moller KL, et al (2016) Changes in Skeletal Integrity and Marrow Adiposity during High-Fat Diet and after Weight Loss. Front Endocrinol 7:102. <https://doi.org/10.3389/fendo.2016.00102>

24. Devlin MJ, Robbins A, Cosman MN, et al (2018) Differential effects of high fat diet and diet-induced obesity on skeletal acquisition in female C57BL/6J vs. FVB/NJ Mice☆. Bone Reports 8:204–214. <https://doi.org/10.1016/j.bonr.2018.04.003>

25. Picke A-K, Sylow L, Møller LLV, et al (2018) Differential effects of high-fat diet and exercise training on bone and energy metabolism. Bone 116:120–134. <https://doi.org/10.1016/j.bone.2018.07.015>

26. Montalvany-Antonucci CC, Zicker MC, Ferreira AVM, et al (2018) High-fat diet disrupts bone remodeling by inducing local and systemic alterations. J Nutr Biochem 59:93–103. <https://doi.org/10.1016/j.jnutbio.2018.06.006>

27. Silva MJ, Eekhoff JD, Patel T, et al (2019) Effects of High‐Fat Diet and Body Mass on Bone Morphology and Mechanical Properties in 1100 Advanced Intercross Mice. J Bone Miner Res 34:711–725. <https://doi.org/10.1002/jbmr.3648>

28. Mansur SA, Mieczkowska A, Flatt PR, et al (2019) Sitagliptin Alters Bone Composition in High-Fat-Fed Mice. Calcif Tissue Int 104:437–448. <https://doi.org/10.1007/s00223-018-0507-0>

29. Colditz J, Picke A, Hofbauer LC, Rauner M (2020) Contributions of Dickkopf‐1 to Obesity‐Induced Bone Loss and Marrow Adiposity. Jbmr Plus 4:e10364. <https://doi.org/10.1002/jbm4.10364>

30. Cao JJ, Gregoire BR, Michelsen KG, Picklo Sr MJ (2020) Decreasing the Ratio of Dietary Linoleic to α-Linolenic Acid from 10 to 4 by Changing Only the Former Does Not Prevent Adiposity or Bone Deterioration in Obese Mice. J Nutr 150:1370–1378. <https://doi.org/10.1093/jn/nxaa044>

31. Ross DS, Yeh T-H, King S, et al (2021) Distinct Effects of a High Fat Diet on Bone in Skeletally Mature and Developing Male C57BL/6J Mice. Nutrients 13:1666. <https://doi.org/10.3390/nu13051666>

32. LLabre JE, Trujillo R, Sroga GE, et al (2021) Circadian rhythm disruption with high‐fat diet impairs glycemic control and bone quality. Faseb J 35:e21786. <https://doi.org/10.1096/fj.202100610rr>

33. Omer M, Ali H, Orlovskaya N, et al (2022) Omega-9 Modifies Viscoelasticity and Augments Bone Strength and Architecture in a High-Fat Diet-Fed Murine Model. Nutrients 14:3165. <https://doi.org/10.3390/nu14153165>

34. Xia B, Zhu R, Zhang H, et al (2022) Lycopene Improves Bone Quality and Regulates AGE/RAGE/NF-кB Signaling Pathway in High-Fat Diet-Induced Obese Mice. Oxid Med Cell Longev 2022:3697067. <https://doi.org/10.1155/2022/3697067>

35. LLabre JE, Sroga GE, Tice MJL, Vashishth D (2022) Induction and rescue of skeletal fragility in a high-fat diet mouse model of type 2 diabetes: An in vivo and in vitro approach. Bone 156:116302. <https://doi.org/10.1016/j.bone.2021.116302>

36. Song W, Sheng Q, Bai Y, et al (2022) Obesity, but not high-fat diet, is associated with bone loss that is reversed via CD4+CD25+Foxp3+ Tregs-mediated gut microbiome of non-obese mice. Npj Sci Food 7:14. <https://doi.org/10.1038/s41538-023-00190-6>

37. Beaver LM, Prati M, Gilman KE, et al (2023) Diet composition influences the effect of high fat diets on bone in growing male mice. Bone 176:116888. <https://doi.org/10.1016/j.bone.2023.116888>

38. Buckels EJ, Tan J, Hsu H, et al (2023) Preptin Deficiency Does Not Protect against High‐Fat Diet‐Induced Metabolic Dysfunction or Bone Loss in Mice. JBMR Plus 7:e10777. <https://doi.org/10.1002/jbm4.10777>
